# Pou3f4-Mediated Regulation of *Ephrin-B2* Controls Temporal Bone Development in the Mouse

**DOI:** 10.1371/journal.pone.0109043

**Published:** 2014-10-09

**Authors:** Steven Raft, Thomas M. Coate, Matthew W. Kelley, E. Bryan Crenshaw, Doris K. Wu

**Affiliations:** 1 Section on Sensory Cell Regeneration and Development, National Institute on Deafness and Other Communication Disorders, National Institutes of Health, Bethesda, Maryland, United States of America; 2 Laboratory of Cochlear Development, National Institute on Deafness and Other Communication Disorders, National Institutes of Health, Bethesda, Maryland, United States of America; 3 Children's Hospital of Philadelphia, University of Pennsylvania, Philadelphia, Pennsylvania, United States of America; University of Massachusetts Medical, United States of America

## Abstract

The temporal bone encases conductive and sensorineural elements of the ear. Mutations of *POU3F4* are associated with unique temporal bone abnormalities and X-linked mixed deafness (DFNX2/DFN3). However, the target genes and developmental processes controlled by POU3F4 transcription factor activity have remained largely uncharacterized. Ephrin-B2 (Efnb2) is a signaling molecule with well-documented effects on cell adhesion, proliferation, and migration. Our analyses of targeted mouse mutants revealed that *Efnb2* loss-of-function phenocopies temporal bone abnormalities of *Pou3f4* hemizygous null neonates: qualitatively identical malformations of the stapes, styloid process, internal auditory canal, and cochlear capsule were present in both mutants. Using failed/insufficient separation of the stapes and styloid process as a quantitative trait, we found that single gene *Efnb2* loss-of-function and compound *Pou3f4*/*Efnb2* loss-of-function caused a more severe phenotype than single gene *Pou3f4* loss-of-function. *Pou3f4* and *Efnb2* gene expression domains overlapped at the site of impending stapes-styloid process separation and at subcapsular mesenchyme surrounding the cochlea; at both these sites, *Efnb2* expression was attenuated in *Pou3f4* hemizygous null mutants relative to control. Results of immunoprecipitation experiments using chromatin isolated from nascent middle ear mesenchyme supported the hypothesis of a physical association between Pou3f4 and specific non-coding sequence of *Efnb2*. We propose that *Efnb2* is a target of Pou3f4 transcription factor activity and an effector of mesenchymal patterning during temporal bone development.

## Introduction

**Figure 1:**
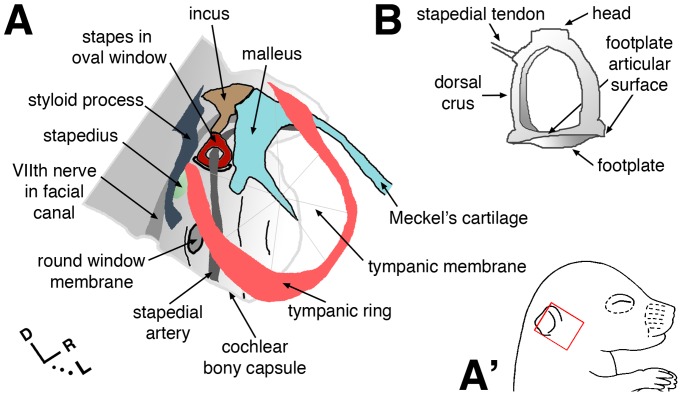
Schematic views of selected middle ear structures and otic capsule. (**A**) Anatomy conforms to that of an excised mouse temporal bone at post-natal day 5, viewed laterally. Orientation within the head is indicated by the red box in A′, bottom right. R in axes  =  rostral. (**B**) Caudal view of the isolated adult stapes.

Paired temporal bones at the sides and base of the skull house conductive and sensori-neural components of the peripheral auditory system [1]. The tympanic part (middle ear) is air-filled and contains structures that conduct sound energy to the cochlea: the tympanic membrane, auditory ossicles (malleus, incus, stapes), muscles, and ligaments (Fig. 1A). The petrous portion of the temporal bone (bony capsule) encases inner ear sensory end organs and neuronal ganglia within a labyrinthine space, and forms canals for passage of cranial nerves and major vessels. It protects the auditory-vestibular system and contributes to the architecture of partitioned, fluid-filled spaces that comprise the inner ear.

Various parts of the temporal bone form by either endochondral or intramembranous ossification [2]. The auditory ossicles and petrous portion form by endochondral ossification, and in mice, these structures are cartilaginous at the time of birth. The petrous portion and its developmental precursor (cartilaginous otic capsule) derive from head mesoderm [3]. The distal ossicles (malleus and incus) derive from cranial neural crest [4]. The stapes is of mixed origin [5]; head and crura derive exclusively from neural crest, and the footplate is a mixed derivative of neural crest and head mesoderm (Fig. 1B).

Genetic classification of otologic conditions and identification of pathogenic gene mutations provide insights into temporal bone development. X-linked mixed (conductive and sensorineural) progressive deafness with stapes fixation and perilymphatic gusher (a profuse discharge of inner ear and cerebrospinal fluid) correlates with unique temporal bone abnormalities (cochlear hypoplasia; dilated internal auditory canal) upon radiological examination [6]–[9]. The genetic locus for this non-syndromic form of deafness (DFNX2, formerly DFN3; OMIM 304400) maps to Xq21.1 [10] and is associated with mutations of coding or regulatory sequence for the *POU3F4* gene [11], [12]. POU family transcription factors bind to DNA in a sequence-specific manner through the bipartite motif of POU homeodomain and POU-specific domains [13]. In the mouse, *Pou3f4* is expressed in mesenchyme surrounding the embryonic inner ear epithelium, and targeted mutagenesis of *Pou3f4* causes multiple developmental malformations of the ear, including cochlear hypoplasia, thin cochlear bony capsule (petrous temporal bone), dilated internal auditory canal, and malformed stapes [14]–[16]. Mice lacking *Pou3f4* also show abnormalities of specialized fibrocytes apposed to the bony cochlear wall, a dramatically reduced endolymph potential, and profound deafness, suggesting that loss of *Pou3f4* disrupts cochlear fluid homeostasis [17], [18]. However, very little is known about target genes that mediate the effects of *Pou3f4* on ear development.

*Ephrin-B2* (*Efnb2*) encodes a Type I transmembrane protein that functions as a cell contact-dependent ligand for multiple Eph receptor tyrosine kinases. Upon binding to a cognate Eph protein, Efnb2 can also function cell-autonomously by signaling from its cytoplasmic domain through SH2 and PDZ domain-containing adaptor proteins [19], [20]. Eph/ephrin-B signaling influences cell migration, progenitor cell sorting, and tissue boundary formation in the developing embryo through effects on cytoskeletal organization and adhesion between adjacent cells [21], [22]. Eph/ephrin-B signaling also controls cell proliferation in the embryo and adult stem cell niches of mice [23]–[27]. Recent evidence indicates that Pou3f4 transcription factor regulates Eph-ephrin signaling: Pou3f4 in mesenchyme surrounding the cochlea binds to specific non-coding sequence of *Epha4* and is necessary for proper expression of *Epha4* [28], the product of which is a canonical receptor for Efnb2 [21]. This regulation is associated with proper fasciculation of axons that innervate the cochlea [28]. Here, we report on temporal bone morphologies of single and compound *Pou3f4* and *Efnb2* loss-of-function mutants, developmental gene expression in wild type and mutant embryos, and anti-Pou3f4-mediated pull-down of *Efnb2* sequence from chromatin of the embryonic ear. The results provide evidence that *Efnb2* is a direct effector of Pou3f4 activity during temporal bone development.

## Results

### Adult *Pou3f4* ^Cre/Y^ mice exhibit defects of the stapes, facial canal, internal auditory canal, and bony capsule of the cochlea

**Figure 2:**
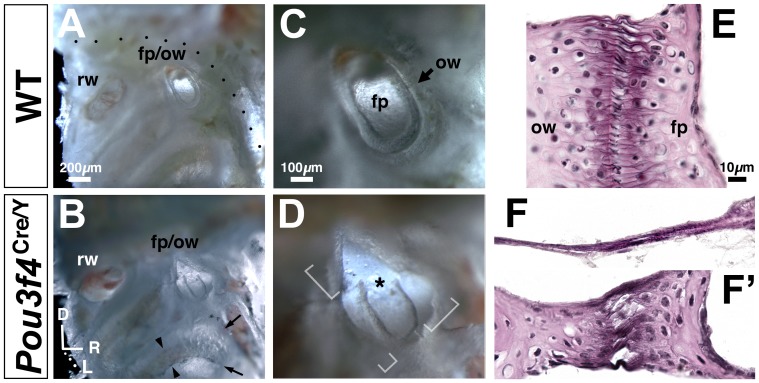
Abnormalities of the stapedio-vestibular (S–V) joint and cochlear bony capsule in *Pou3f4* ^Cre/Y^ males. (**A–D**) Views of the middle ear medial wall/cochlear bony capsule from control (A,C) and *Pou3f4* ^Cre/Y^ temporal bones (B,D) at 16 weeks of age. Soft tissue and bony structures, including the stapes head and crura, are dissected away for unobstructed views of the stapes footplate (fp) in oval window (ow) and cochlear bony capsule. rw, round window. (C,D) are magnified views of the fooplate/oval window in (A,B), respectively. Brackets in (D) highlight disarticulation at a severely affected mutant S–V joint with hypoplastic footplate. Asterisk highlights artifactual fracture of the footplate during dissection. Dotted line in (A) shows the course of the VIIth nerve within the facial canal. Arrows in (B) highlight translucence of the thin mutant cochlear bony capsule compared to wild-type; arrowheads in (B) highlight visibility of strial blood vessels and melanocytes through the mutant bony capsule. Axes in (B) apply to (A–D). (**E, F, F′**) Wild-type (E) and mutant (F, F′) S–V joint in histological section stained for elastic fibers (black), collagen (light pink), and nuclei. (F) corresponds to a bracketed regions of disarticulation in (D); (F′) corresponds to a region of mutant S–V joint where footplate and oval window are more closely apposed. Corresponding photos of wild-type and mutant are shown to scale.

Mutant mice wherein *cre* replaces the coding region of *Pou3f4* (resulting in a null allele of *Pou3f4*) were previously characterized with respect to spiral ganglion development [28]. We analyzed excised temporal bones from 16–20 week old *Pou3f4* ^Cre/Y^ hemizygous null males (n = 40) and wild-type male littermate controls (n = 62). The stapedio-vestibular (S–V) joint, which forms a functional interface between middle and inner ear chambers, comprises articular surfaces of the stapes footplate and oval window of the inner ear bony capsule (Fig. 1A, B). At the S–V joint of wild-type controls, articular surfaces were closely apposed and joined by elastic fibers; collectively, these fibers form the annular ligament (Fig. 2A, C, E) [29]. By contrast, all mutant footplates were disarticulated from the oval window to varying degrees (Fig. 2B, D), with both footplate hypoplasia and oval window dysplasia contributing to the defect. Severely affected ‘floating’ footplates (Fig. 2D) were tethered to the oval window by a thin elastin- and collagen-containing membrane (Fig. 2F). Less affected regions of the mutant S-V joint showed disorganized elastic fibers, irregular articular surfaces, and reduced thickness of footplate and oval window bone compared to controls (Fig. 2E, F′). The entire mutant cochlear bony labyrinth was abnormally thin (Fig. 2A, B; arrows) and fragile upon dissection.

**Figure 3:**
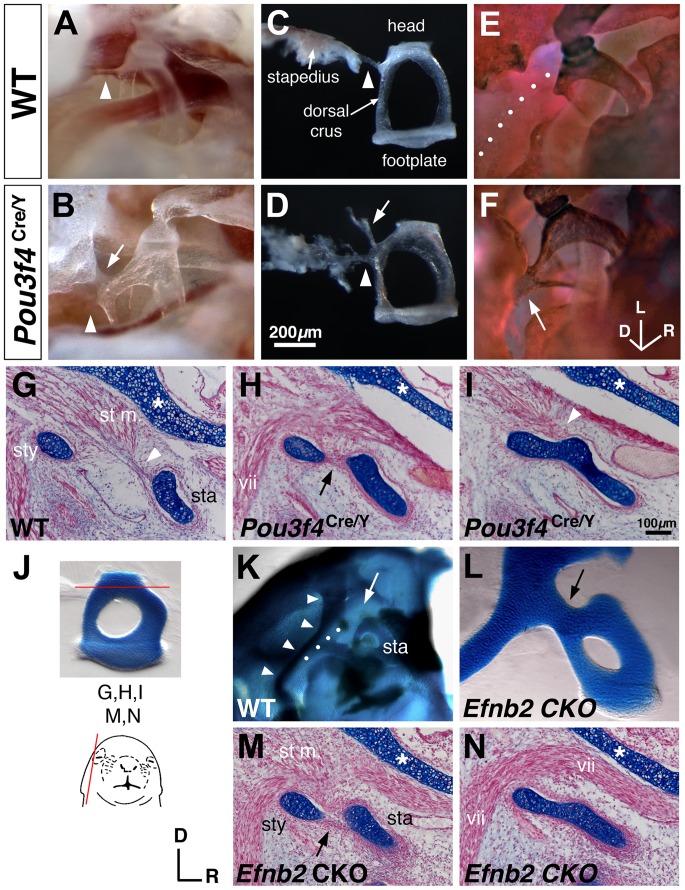
Stapes coalition in *Pou3f4* ^Cre/Y^ and Sox9-IRES-Cre-mediated *Efnb2* CKO mice. (**A–D**) Unstained preparations of the stapes in situ (A, B) and dissected (C, D) from *Pou3f4* ^+/Y^ wild-type (A, C) and *Pou3f4* ^Cre/Y^ mutant (B, D) males at 16 weeks of age. Arrowheads highlight the stapedial tendon. Arrows in (B, D) highlight a periosteal soft tissue bridge connecting the dorsal crus of the stapes and facial canal. (**E, F**) Alizarin red/alcian blue-stained preparation of wild-type (E) and mutant (F) stapes in situ at 16 weeks of age, showing a bony fusion of the dorsal crus to the facial canal in a mutant (arrow, F). Dotted line in (E) highlights the facial nerve. (**G–I**) Alcian blue/fast red-stained sagittal sections of P1 wild-type (G) and *Pou3f4* mutants (H, I) at the level of the stapes head (sta), stapedius muscle (st m.), styloid process (sty), and facial nerve (vii). Arrow in (H) highlights a continuous perichondrium enveloping both the styloid process and the stapes. Arrowheads highlight insertion of the stapedial tendon into the stapes perichondrium. Asterisks highlight the cochlear capsule. (**J**) Photo of a dissected P0 wild-type stapes showing the level of section for (G,H,I,M,N). Schematic shows the plane of section. (**K, L**) Alizarin red/alcian blue-stained preparations from E19 wild-type (K) or *Efnb2* CKO fetuses. (K) shows the wild-type otic capsule with middle ear structures dissected away for unobstructed view of the stapes (sta) and styloid process (arrowheads). White arrow highlights the lack of connection between normal stapes and styloid process. Dotted line hightlights a segment of the facial canal. (L) shows a magnified (relative to K) view of a dissected mutant stapes and styloid process; black arrow highlights the abnormal connection between these structures. (**M, N**) Alcian blue/fast red-stained sagittal sections from two different E19 *Efnb2* CKO heads, showing full cartilage coalition of stapes and styloid process (N) or reduced distance/shared perichondrium between the two structures. Level of sections is comparable to (H, I). Axes near (M) and scale bar in (I) apply to all histological sections. Axes in (F) apply to (A, B, E, F, K, L). Scale bar in (D) applies to (A–F).

The facial canal is a bony sulcus running along the dorsal aspect of the middle ear cavity; it contains the stapedius muscle and a segment of the VIIth cranial nerve (Fig. 1A), tissues that mediate the acoustic reflex and modify stapes mobility. Compared to controls, *Pou3f4* ^Cre/Y^ mutant stapes were shifted dorsally and set more closely to the facial canal (data not shown). In 45% of mutant ears analyzed (18/40), a thin sheath of periostium connected the dorsal crus of the stapes to an abnormal prominence on the lateral wall of the facial canal (Fig. 3A–D); this soft tissue bridge always co-occurred with an intact stapedial tendon and did not grossly limit stapes mobility as assessed by palpation. In 33% of mutant ears analyzed (13/40), this same region of the dorsal crus was fused to the lateral wall of the facial canal by a bone bridge (Fig. 3E, F), causing an immobile stapes. All mutant ears with a bony coalition lacked the stapedial tendon.

To assess the validity of scoring individual temporal bones in generating data sets (denoted as ‘occurrence’ of a defect, where *n* =  the number of temporal bone specimens), we analyzed phenotypic penetrance by tabulating the prevalence of every possible combination of stapes-facial canal connections (unilateral vs. bilateral; soft tissue vs. bone) in the sample of *Pou3f4* ^Cre/Y^ mutants (*n* =  the number of mutant animals sampled; Table S1). The distribution did not deviate significantly from the case where each category is equally prevalent (P = 0.6526, Chi-square 3.308; 5 df). This supports the assumption that stapes-facial canal connection type (soft or bone) and unilaterality/bilaterality of the defect within an individual are independent events. No left- or right-sided bias was noted among animals with a unilateral defect.

At the medial side of the temporal bone, the internal auditory canal comprises three foramina for passage of VIIth and VIIIth cranial nerve branches between the bony labyrinth and brain. *Pou3f4* null hemizygosity caused incomplete septation and dilation of these foramina (Fig. S1A, B), as previously shown [14]. Together, these results extend previous characterizations [14], [15], [30] of bony defects in the *Pou3f4* mutant ear.

### Most temporal bone defects of *Pou3f4* ^Cre/Y^ mice are evident at the endochondral cartilage stage of development

We next investigated the developmental origins of *Pou3f4* ^Cre/Y^ temporal bone defects. Analyses of normally developing middle ears between post-natal days 5 and 15 indicated that the lateral wall of the facial canal is formed by fusion and growth of the endochondral styloid process and intramembranous tympanic ring (Fig. 1, S2). At perinatal stages, the styloid process is a rod of cartilage extending from the dorso-lateral aspect of the inner ear capsule (Fig. 3K, arrowheads; Fig S2A, D, G). To determine whether bony fixation of the stapes and facial canal in *Pou3f4* ^Cre/Y^ adults is caused by ossification of an abnormal cartilage rudiment, we analyzed serial sections of *Pou3f4* ^Cre/Y^ (n = 22) and wild-type (n = 17) temporal bones from E18.5-P0 littermates. All wild-type ossicles were discrete, cartilaginous structures enveloped by perichondrium and surrounded by loose mesenchyme or a developing joint space (Fig. 3G, J). However, in 36% of perinatal *Pou3f4* ^Cre/Y^ ears analyzed (8/22), the cartilaginous dorsal crus and styloid process were connected (Fig. 3I), and the stapedius muscle and tendon were present but dysmorphic (Fig. 3G, I, arrowheads). In 32% of the perinatal mutant ears analyzed (7/22), distance between the unconnected stapes and styloid process was markedly reduced compared to control and a common perichondrium enveloped both structures (Fig. 3H, arrow). Thus, the *Pou3f4* ^Cre/Y^ perinatal-stage phenotype can be categorized as a partially or fully coalesced stapes and styloid process. The relative occurrence of these defects in our perinatal stage sample did not differ from the relative occurrence of soft and bone bridges identified in adult mutants (Table 1; Chi-square 1.152; df, 2; P = .5622; also see Table S1). Thus, the soft tissue bridge and bone bridge abnormalities identified in adult *Pou3f4* ^Cre/Y^ mice are likely consequences of perinatal ‘close proximity/common perichondrium’ and ‘continuous cartilage’ abnormalities, respectively. Other malformations evident in neonatal *Pou3f4* ^Cre/Y^ mutants included a thin cochlear cartilage capsule (Fig. 3G–I, asterisks) and dilation/incomplete septation of the internal auditory canal (Fig. S1D, E). However, we found no perinatal cartilage abnormality that might account for S–V joint disarticulation observed in adult mutants (data not shown). Thus, a subset of *Pou3f4* ^Cre/Y^ temporal bone malformations is manifested at the cartilaginous stage of endochondral bone formation. These results are consistent with immunohistochemical evidence of Pou3f4 activity during early phases of periotic mesenchymal patterning and/or condensation [31].

**Table 1:** Occurrence of stapes-facial canal or -styloid connectivity in *Pou3f4* ^Cre/Y^ and *Efnb2* CKO mice.

Genotype/Stage	*n* a	Soft Tissue Bridge	Bone or Cartilage Bridge	Total Affected
*Pou3f4* ^Cre/Y^ adult	40	18 (.45)	13 (.33)	31 (.78)
*Pou3f4* ^Cre/Y^ neonate	22	7 (.32)	8 (.36)	15 (.68)
*Efnb2* CKO fetal (E19)	20	2 (.10)	14 * (.70)	16 (.80)

a sample size refers to the number of ears analyzed.

* P = 0.0365, compared to *Pou3f4* ^Cre/Y^ neonate by Fisher's exact test. Statistical significance set at P<0.05.

### *Efnb2* and *Pou3f4* mRNA signals overlap in mesenchyme surrounding the VIIth nerve, condensing stapes, spiral ganglion, and cochlear duct

**Figure 4:**
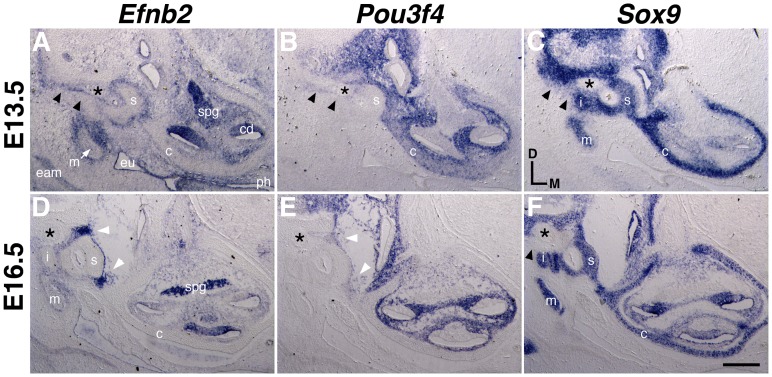
Comparative expression of *Efnb2*, *Pou3f4*, and *Sox9* at E13.5 and E16.5. Adjacent transverse sections through embryos at two developmental stages, hybridized to detect *Efnb2* (A,D), *Pou3f4* (B, E), or Sox9 (C, F). *c*, cartilaginous cochlear capsule; *cd*, cochlear duct; *eam*, external auditory canal; *eu*, nascent eustacian tube; *i*, incus; *m*, malleus; *ph*, pharynx, *spg*, spiral ganglion, *s*, stapes. Asterisk highlights the facial nerve. Double arrowheads in (A–C) highlight overlapping *Efnb2* and *Pou3f4* signals and relatively weak *Sox9* signal dorsal to the stapes. Single arrowhead in (F) highlights a lack of Sox9 signal in the corresponding region at E16.5. White arrowheads in (D, E) highlight overlapping *Efnb2* and *Pou3f4* signals at the forming S–V joint. Scale bar represents 200 microns in (A–C) and 250 microns in (D–F).

Periotic mesenchymal patterning and condensation in the mouse are evident between stages E11 and E13.5 [32], and we identified *Efnb2* mRNA surrounding periotic mesenchymal condensations during these and later stages of development (Fig. 4A, D). At E13.5, *Efnb2* and *Pou3f4* mRNA expression patterns in the developing middle ear were complementary and partially overlapping. Overlap was noted in mesenchyme apposed to the stapes footplate, within the developing S–V joint, dorsal to the stapes, and surrounding the VIIth nerve (Fig. 4A, B). *Efnb2* and *Pou3f4* signals also overlapped in sub-capsular mesenchyme surrounding the cochlear duct and spiral ganglion, as previously shown [28]. *Efnb2* and *Pou3f4* expression was complementary across the lateral wall of the cochlear capsule (Fig. 4A, B; denoted by c). As previously shown [14], [28], [31], *Pou3f4* was not expressed in the developing inner ear epithelium, spiral ganglion, or mesenchyme surrounding the incus and malleus, all of which express *Efnb2*.

**Figure 5:**
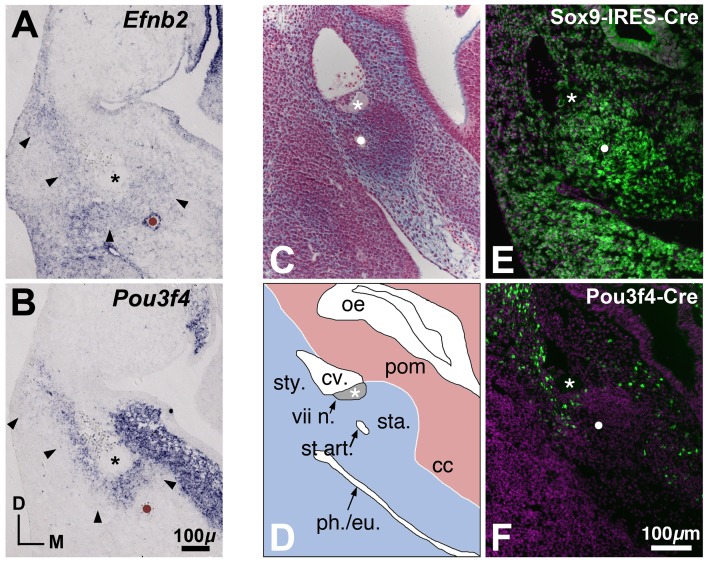
Comparative gene and Cre-mediated reporter expression in the developing middle ear at E12.5. (**A, B**) *Efnb2* and *Pou3f4* gene expression domains, as shown in adjacent transverse sections through a wild-type E12.5 embryo. The stapedial artery, positioned roughly at the center of the stapes condensation, is marked by brown dots and by strong expression of *Efnb2* in the arterial endothelium (A). Asterisks highlight the facial nerve dorsal to the stapes condensation. Arrowheads highlight a region of overlapping *Efnb2* and *Pou3f4* signal dorsal to the stapedial artery and surrounding the facial nerve. (**C, D**) Transverse section through a wild-type E12.5 embryo stained with alcian blue/nuclear fast red (C) and a scaled schematic drawing (D) of the stapes condensation and neighboring structures. White dot in (C) highlights the stapedial artery. Asterisks in (C,D) highlight the VIIth nerve. *cc*, otic capsule condensation; *cv*., cardinal vein; *oe*, otic epithelium; *ph./eu*., nascent pharynx/eustachian tube; *pom*, periotic mesenchyme; *sta*, stapes mesenchymal condensation; st art., stapedial artery; *sty*, styloid process mesenchymal condensation; *vii n*., VIIth nerve. (**E, F**) Sox9-IRES-Cre- (E) and Pou3f4-Cre- (F) mediated ROSA-YFP reporter expression in the region of the E12.5 nascent middle ear, as schematized in (D). (C–F) are shown to scale. Cre-positive cells or descendents of Cre-positive cells are labeled green. DAPI-positive nuclei are purple. White dots highlight the stapedial artery; asterisks highlight the VIIth nerve.

By RNA hybridization, we first detected *Efnb2* signal in the nascent middle ear between E11.5 and E12.5; at E12.5, *Efnb2* and *Pou3f4* signals overlapped in branchial arch mesenchyme dorsal to the stapedial artery and surrounding the VIIth cranial nerve (Fig. 5A, B). At E16.5, *Pou3f4* signal intensity in mesenchyme surrounding the stapes had decreased relative to earlier stages, whereas *Efnb2* signal within the developing S–V joint had intensified relative to earlier stages (Fig. 4A, B, D, E); conversely, *Efnb2* signal in E16.5 cochlear subcapsular mesenchyme was weak compared to earlier stages, whereas *Pou3f4* signal remained robust in mesenchyme surrounding the E16.5 cochlea and spiral ganglion.

In summary, *Efnb2* and *Pou3f4* mRNA signals show the greatest degree of overlap between stages E12.5 and E13.5, as periotic mesenchyme is patterned and condensed into pre-chondrogenic rudiments (Fig. 5C, D). Overlap of signals between stages E12.5 and E13.5 occurs at sites relevant to the *Pou3f4* ^Cre/Y^ stapes phenotype described above.

### Sox9-IRES-Cre-mediated inactivation of *Efnb2* phenocopies cartilage abnormalities of the perinatal *Pou3f4* ^Cre/Y^ ear

To assess the function of *Efnb2* in ossicular development, we inactivated *Efnb2* broadly in mesenchymal and epithelial components of the ear using the Sox9-IRES-Cre driver (Fig. 5E; S3) [24]. Sox9-IRES-Cre^+^;*Efnb2* ^flox/null^ (*Efnb2* CKO) pups were delivered live, but died within 12 hours of birth. Alcian blue-stained whole skeletal preparations of late-stage *Efnb2* CKO fetuses had no obvious axial, appendicular, or craniofacial deformities. However, of 20 *Efnb2* CKO ears analyzed at E19 by either histological or whole mount cartilage/bone staining, 14 showed complete coalition of the stapes and styloid process and 2 showed reduced distance between stapes and styloid process cartilages and a shared perichondrium (Fig. 3K–N). These phenotypes were morphologically indistinguishable from those of the perinatal *Pou3f4* ^Cre/Y^ male (compare Fig. 3M, N and H, I). The total occurrence of all identified stapes abnormalities in the fetal *Efnb2* CKO sample (Table 1, Total Affected; see also Table S1) did not differ from that of the perinatal *Pou3f4* ^Cre/Y^ sample (P = 0.4913; Fisher's exact test). However, the occurrence of a fully connected stapes-styloid cartilage bridge phenotype in the fetal *Efnb2* CKO sample was increased over that of the perinatal *Pou3f4* ^Cre/Y^ male by roughly 2-fold (P = 0.0365; Fisher's exact test). Other endochondral cartilage-stage temporal bone defects observed in both *Efnb2* CKO and Pou3f4^Cre/Y^ mutants included a thin cochlear cartilaginous capsule compared to control (Fig. 3G, H, I, M, N, asterisks) and dilation/incomplete septation of the internal auditory canal (Fig. S1D–F). As with neonatal *Pou3f4* ^Cre/Y^ mice, we found no abnormality at the developing S–V joint in stage E19 *Efnb2* CKO fetuses (data not shown). Distal ossicles (incus and malleus) in the *Efnb2* CKO were judged to be of normal size and morphology. Together, these results indicate that Sox9-IRES-Cre-mediated loss of *Efnb2* phenocopies cartilage abnormalities of the perinatal *Pou3f4* ^Cre/Y^ ear.

We next analyzed a sample of E19 fetuses homozygous for an Efnb2 C-terminal truncation (ephrin-B2^LacZ^ mice in [33]–[35]). This allele produces an Efnb2:ß-galactosidase fusion protein that is targeted to the plasma membrane and binds cognate EphB receptors on adjacent cells, but is unable to transduce signals to the cytoplasmic side of the membrane; thus, it can provide evidence of whether a phenotype is due to a specific loss of (forward) signaling through an Eph receptor or (reverse) signaling through the Efnb2 C-terminus. Histological analyses of 7 ears from 5 *Efnb2* C-terminal deletion homozygotes revealed neither a coalesced stapes-styloid process nor reduced distance between the two structures, and other components of the developing middle ear appeared normal (Fig. S4). These results suggest that the Efnb2 C-terminus is dispensable in forming separate stapes and styloid process cartilages during development.

### Compound loss of *Efnb2* and *Pou3f4* potentiates the *Pou3f4* ^Cre/Y^ adult stapes phenotype

Recapitulation and increased severity of the perinatal *Pou3f4* ^Cre/Y^ stapes-styloid defect in Sox9-IRES-Cre^+^;*Efnb2* ^flox/null^ E19 fetuses prompted us to ask whether *Pou3f4* ^Cre/Y^;*Efnb2* ^flox/null^ compound mutant mice are viable and exhibit a more severe middle ear phenotype than that of *Pou3f4* ^Cre/Y^ adult mice. We therefore generated and analyzed the series of compound mutant phenotypes listed in Table 2. Analyses of Pou3f4-Cre;ROSA-YFP mice revealed that this cross should inactivate the floxed *Efnb2* allele in subcapsular cochlear mesenchyme, mesenchyme within the forming S–V joint, and branchial arch mesenchyme dorsal to the stapes and surrounding the VIIth nerve (Fig. 5F; data not shown). Pou3f4-Cre-mediated *Efnb2* CKO mice were viable through at least 6 months of age. We therefore analyzed the ears of 16–20 week old adult male mice. This allowed for rapid categorization of the stapes phenotype as either a soft tissue bridge or bone bridge, as well as an assessment of the S-V joint and internal auditory canal. All compound mutants were of the same mixed genetic background as the *Pou3f4* ^Cre/Y^ males described above (see Materials and Methods, Animals).

**Table 2:** Occurrence of stapes-facial canal connectivity in adult *Pou3f4* and *Efnb2* single and compound mutant mice.

Genotype	*n* a	Soft Tissue Bridge	Bone Bridge	Total Affected
*Pou3f4* ^Cre/Y^; *Efnb2* ^+/+^	40	18 (.45)	13 (.33)	31 (.78)
*Pou3f4* ^Cre/Y^; *Efnb2* ^+/flox^	38	9 (.24)	13 (.34)	22 (.57)
*Pou3f4* ^Cre/Y^; *Efnb2* ^null/+^	32	10 (.31)	8 (.25)	18 (.56)
*Pou3f4* ^Cre/Y^; *Efnb2* ^null/flox^	39	1** (.03)	23* (.59)	24 (.62)
*Pou3f4* ^+/Y^; *Efnb2* ^null/+^	30	0	0	0
*Pou3f4* ^+/Y^; *Efnb2* ^+/+^	62	0	0	0

a sample size refers to the number of ears analyzed.

* 0.02<P<0.05;

** p<0.0001; all pairwise Fisher's exact tests were performed relative to the *Pou3f4* ^Cre/Y^; *Efnb2* ^+/+^ genotype. Statistical significance set at P<0.05.

Table 2 shows genotypes and categorical data for the stapes-facial canal abnormality. No such abnormalities were observed in a sample of *Efnb2* null heterozygote temporal bones (*Pou3f4* ^+/Y^; *Efnb2* ^null/+^ or *Pou3f4* ^+/Y^; *Efnb2* ^null/flox^, n = 30), so these genotypes were excluded from further analyses. A Chi-square test of independence on the remaining data shown in Table 2 indicated that the variables of genotype and phenotype are related (Chi-square  = 23.82; df = 6; P = 0.0006). Pair-wise comparisons between *Pou3f4* ^Cre/Y^; *Efnb2* ^+/+^ and *Pou3F4* ^Cre/Y^; *Efnb2* ^+/flox^ or *Pou3F4* ^Cre/Y^; *Efnb2* ^null/+^ genotypes revealed no differences in occurrence for any category (soft tissue bridge; bone bridge; and total affected, ie., soft + bony connections), indicating that *Efnb2* heterozygosity does not potentiate the *Pou3f4* hemizygous null stapes-facial canal phenotype. Comparisons between *Pou3f4* ^Cre/Y^;*Efnb2* ^+/+^ and *Pou3f4* ^Cre/Y^;*Efnb2* ^null/flox^ genotypes revealed that the total occurrence of affected stapes (Total Affected) was similar across genotypes (P = 0.1471; Fisher's exact test). However, the sample of mice lacking both genes had a roughly 2-fold increase in occurrence of bony connections over that of *Pou3f4* ^Cre/Y^ hemizygous null males (P = 0.0243; Fisher's exact test) and a decrease in soft tissue connections compared to *Pou3f4* ^Cre/Y^ hemizygous null males (P <0.0001; Fisher's exact test). In analyzing penetrance of phenotypic categories for the *Pou3f4* ^Cre/Y^;*Efnb2* ^null/flox^ sample, we found that the distribution for all six possible categories does deviate from the case where each category is equally prevalent (P = 0.0419, Chi-square: 11.53, 5 df), but bilateral and unilateral bony defects (which dominate the distribution) are equally prevalent in the sample (P >0.7232, by Chi-square or Fisher's exact test; Table S1). In summary, compound *Pou3f4* null hemizygosity;*Efnb2* conditional null homozygosity biases expressivity of the *Pou3f4* ^Cre/Y^ stapes-facial canal defect toward the more severe form (bone bridge) independently of its bilateral or unilateral configuration. Severity of defects at the S–V joint and internal auditory canal were judged to be similar across *Pou3f4* ^Cre/Y^;*Efnb2* ^+/+^ and *Pou3f4* ^Cre/Y^;*Efnb2* ^null/flox^ genotypes (Fig. S1B, C; data not shown).

### Eph receptor gene expression foreshadows borders of distinct middle ear structures

As described in a previous section, we found no stapes-styloid defects in a small sample of Efnb2 C-terminal deletion mutants, so Efnb2 may function as a ligand to activate Eph receptor (forward) signaling [19]–[21] in the present context. We surveyed the middle ear rudiment for expression of *Ephb1*, *Ephb2*, *Ephb3*, *Ephb4*, and *Epha4*, each of which encodes a cognate receptor for Efnb2. *Sox9* hybridization signal, a marker of osteo-chondroprogenitor cells [36], was used in conjunction with histological appearance and anatomical landmarks to identify territories of nascent middle ear rudiments. Of the set of Eph receptor genes tested, *Ephb4* was expressed widely throughout developing middle ear mesenchyme between E11.5 and E13.5 (data not shown), whereas *Epha4* and *Ephb2* were expressed in patterns that foreshadow borders of distinct middle ear structures.

**Figure 6:**
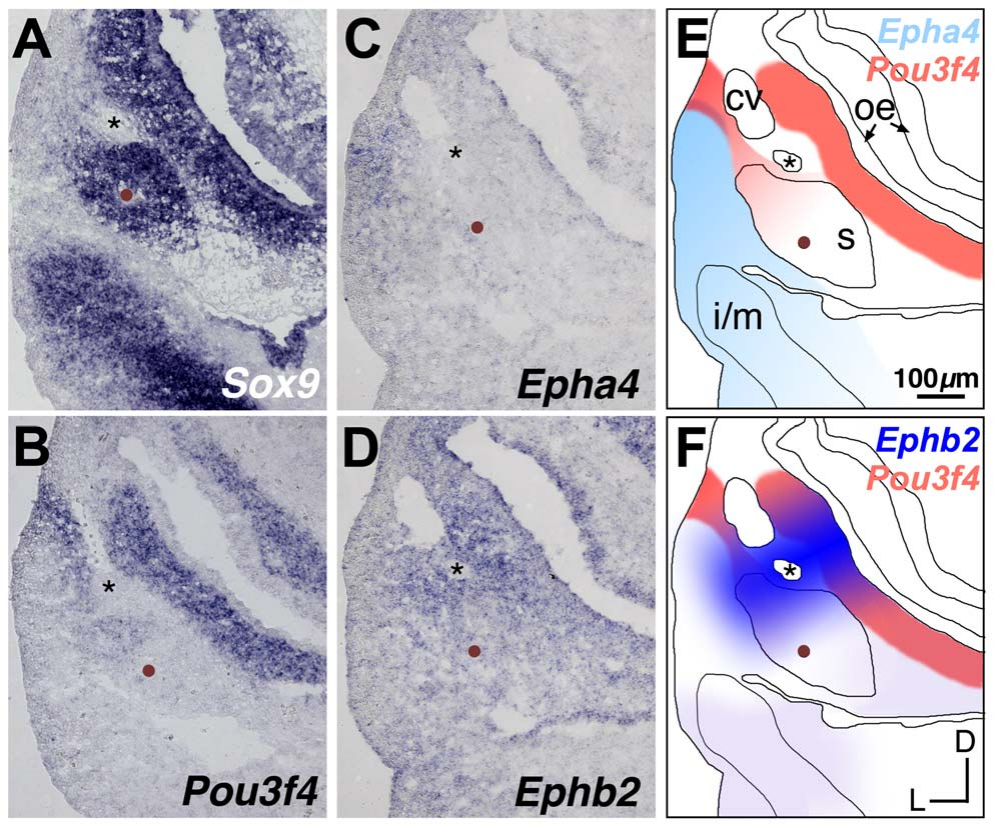
Comparative expression of *Sox9*, *Pou3f4*, *Epha4*, and *Ephb2* at E11.5. (**A–D**) Adjacent transverse sections through a wild-type E11.5 embryo, hybridized to detect *Sox9, Pou3f4, Epha4*, or *Ephb2*. Mesenchyme that will form the stapes condensation is identified by *Sox9* signal surrounding the stapedial artery (brown dots) and is located ventral to the facial nerve (asterisks). (**E, F**) Schematized spatial relationships between expression of *Pou3f4* and either *Epha4* (E) or *Ephb2* (F). Asterisks denote VIIth nerve; brown dots denote the stapedial artery. *cv*, cardinal vein; *oe*, otic epithelium; *s*, stapes pre-condensation; *i/m*, incus/malleus pre-condensation. All panels are shown to scale.

**Figure 7:**
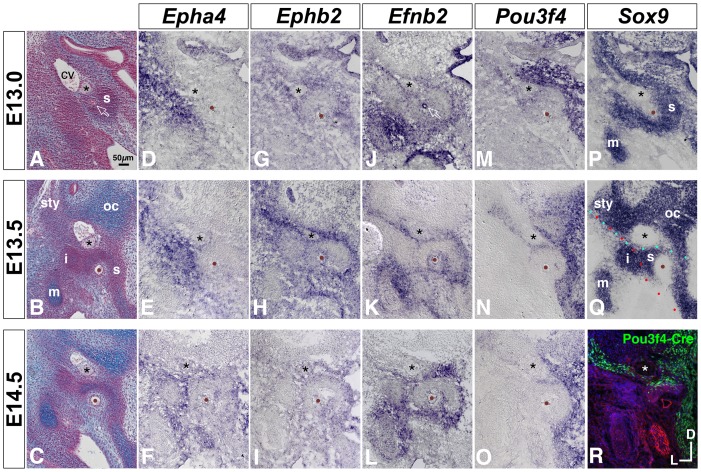
Comparative developmental expression of *Epha4, Ephb2, Efnb2, Pou3f4*, and *Sox9* at the stapes condensation and neighboring structures. (**A–C**) Transverse histological sections through wild-type embryos at E13, E13.5, and E14.5, stained with alcian blue/nuclear fast red. Asterisks highlight the VIIth nerve; brown dots or open arrow in (A) highlight the stapedial artery. cv, cardinal vein; s, stapes condensation; sty, styloid process condensation; oc, otic capsule cartilage; i, incus condensation; m, malleus cartilage. (**D–Q**) Developmental gene expression in wild-type embryos. Annotations are as in (A–C). Red and cyan dotted lines in (Q) highlight salient borders of *Epha4* and *Ephb2*/*Efnb2*/*Pou3f4* expression, respectively. (**R**) Pou3f4-Cre-mediated ROSA-YFP reporter expression at E14.5. Cre-positive cells or descendents of Cre-positive cells are labeled green. Section is counterstained with phalloidin (red) and DAPI (blue). All photos are shown to scale.

At E11.5, *Epha4* mRNA signal marked a lateral region of the nascent middle ear and appeared to overlap slightly with *Pou3f4* signal in mesenchyme adjacent to the anterior cardinal vein (Fig. 6A–C, E). By E13, *Epha4* signal had intensified lateral to the stapes and VIIth nerve and formed a domain complementary to that of *Pou3f4* (Fig. 7A, D, M, P). By E14.5, *Epha4* signal was detected in loose mesenchyme surrounding the ossicular condensations (Fig. 7C, F).

At E11.5, *Ephb2* mRNA signal marked a medial region of the nascent middle ear, with strong signal in mesenchyme dorsal to the stapedial artery and surrounding the VIIth cranial nerve; this overlapped with a portion of *Pou3f4* signal (Fig. 6B, D, F). By E13.5, strong *Ephb2* signal surrounding the VIIth nerve had resolved to a medial-lateral coursing stripe just dorsal to the stapes. The *Ephb2* stripe overlapped with *Pou3f4* and *Efnb2* signals at E13.5 (Fig. 7H, K, N), prior to detectable cartilage matrix deposition in the stapes condensation (Fig. 7B, C). At E14.5, *Ephb2* and *Pou3f4* signals dorsal to the stapes were attenuated relative to earlier stages (Fig. 7I, O). Pou3f4-Cre;ROSA-YFP genetic labeling at E14.5 recapitulated the medial-lateral coursing stripe dorsal to the stapes (Fig. 7R).

In summary, *Epha4* mRNA signal distinguishes lateral middle ear rudiments (incus, malleus) from the more medially situated stapes, VIIth nerve, and cardinal vein; at E13.5, the *Epha4* domain border defines an apparent plane at which the stapes head and incus will articulate (Fig. 7Q, red dots, compare with Fig. 7E). By contrast, *Ephb2* mRNA signal distinguishes the stapes from more dorsally situated rudiments, such as otic capsule surrounding the vestibular canals, VIIth nerve, and styloid process (Fig. 7Q, cyan dots, compare with Fig. 7H).

### *Pou3f4* ^Cre/Y^ null hemizygosity dysregulates *Efnb2* and *Sox9* expression dorsal to the stapes

**Figure 8:**
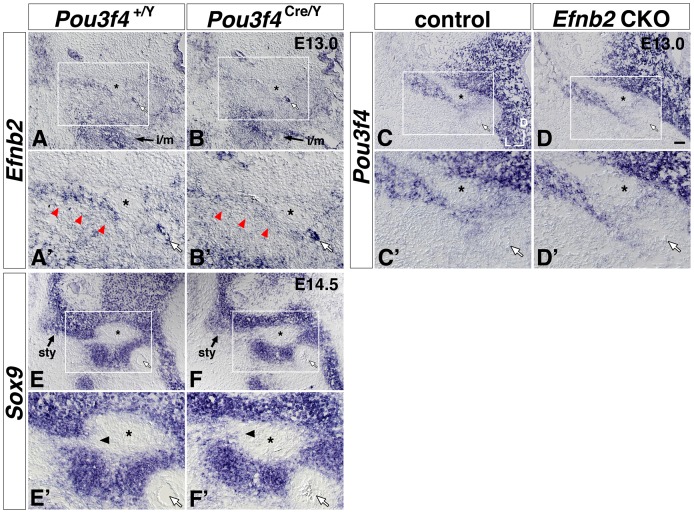
Attenuated *Efnb2* signal and altered patterning of *Sox9* signal near the *Pou3f4* ^Cre/Y^ stapes condensation, but no apparent change of *Pou3f4* signal in the *Efnb2* CKO. (**A, A′, B, B′**) Transverse sections of control (A, A′) and *Pou3f4* ^Cre/Y^ mutant (B, B′) E13.0 littermates hybridized to detect *Efnb2*. Red arrowheads in (A′, B′) highlight the medial-lateral coursing stripe of *Efnb2* signal dorsal to the stapes condensation. i/m, *Efnb2* signal at the incus and malleus rudiment. (**C, C′, D, D′**) Transverse sections of control (C, C′) and Sox9-IRES-Cre-mediated *Efnb2* CKO (D, D′) E13.0 littermates hybridized to detect *Pou3f4*. (**E, E′, F, F′**) Transverse sections of control (E, E′) and *Pou3f4* ^Cre/Y^ mutant (F, F′) E14.5 littermates hybridized to detect *Sox9*. Arrowheads in (E′, F′) highlight an alteration in *Sox9* patterning across genotypes. sty, styloid process condensation. Boxed regions of interest highlighting the stapes and surrounding structures in (A–F) are shown at 2x magnification in (A′–F′), respectively. Scale bar in (D) = 50 micron for (A–F), and 25 microns for (A′–F′). In all panels, white arrows highlight the stapedial artery, and asterisks highlight the VIIIth nerve. Axes in (C) apply to all photos.

The mutant phenotypes described above, together with co-localization of *Ephb2*, *Efnb2*, and *Pou3f4* in a stripe distinguishing the nascent stapes from more dorsally located rudiments, led us to ask whether *Pou3f4* is required for normal expression of *Efnb2, Ephb2, or Epha4* in the developing middle ear. We hybridized serial sections of E13–13.5 *Pou3f4* ^Cre/Y^ mutant and *Pou3f4* ^+/Y^ wild-type littermates with RNA probes and rated the extent and intensity of signals in the region of interest. We found no consistent differences in *Epha4* (4 littermate pairs) or *Ephb2* (6 littermate pairs) signals across genotypes. However, all *Pou3f4* ^Cre/Y^ mutants (from 6 littermate pairs) showed a reduced intensity of *Efnb2* signal immediately dorsal to the stapes and surrounding the VIIth nerve (Fig. 8A, A′, B, B′; S5), as well as in subcapsular mesenchyme surrounding the cochlear epithelium and spiral ganglion (Fig. S6). By contrast, *Efnb2* signals at sites outside the domain of *Pou3f4* expression – the stapedial artery or malleus/incus rudiment, for example - were unchanged across genotypes (Fig. 8A, A′, B, B′; S5). In a converse experiment, we assayed *Pou3f4* expression by RNA hybridization to E13–13.5 *Efnb2* CKO and control embryos (5 littermate pairs) and found no differences in *Pou3f4* signal across genotypes (Fig. 8C, C′,D, D′).

To determine whether attenuated *Efnb2* signal dorsal to the *Pou3f4* ^Cre/Y^ stapes correlates with failed separation of pre-cartilaginous mesenchymal condensations, we assessed *Sox9* hybridization signal in *Pou3f4* ^Cre/Y^ mutant and *Pou3f4* ^+/Y^ wild-type littermate pairs. In E12.5 wild-type embryos, the chondrogenic marker *Sox9* formed a continuous domain comprising Meckel's (1^st^ branchial arch) cartilage, the malleus/incus territory, stapes condensation, otic capsule condensation, styloid process territory, and Reichert's (2^nd^ branchial arch) condensation (Fig. S7). Continuity of *Sox9* expression across the stapes and styloid process condensations was maintained until E13.5 (Fig. 4C, F; black arrowheads), but all E14.5 wild-type embryos (4/4) showed a sharply bordered region of *Sox9* negativity dorsal to the stapes and adjacent to the VIIth nerve (Fig. 8E, E′). E14.5 *Pou3f4* ^Cre/Y^ mutants did not have a sharply bordered region of *Sox9* negativity dorsal to the stapes (0/4); instead, a continuous *Sox9* signal of graded intensity – similar to the *Sox9* pattern in normal E13.5 ears - persisted in the E14.5 mutants (Fig. 8F, F′). In summary, these results are consistent with a model in which *Pou3f4* lies upstream of *Efnb2* in a genetic hierarchy, and attenuated *Efnb2* expression in the *Pou3f4* ^Cre/Y^ mutant is causal for insufficient separation of stapes and styloid process rudiments prior to the onset of local cartilage matrix deposition.

### Pou3f4 protein associates with the *Efnb2* locus in nascent middle ear mesenchyme

**Figure 9:**
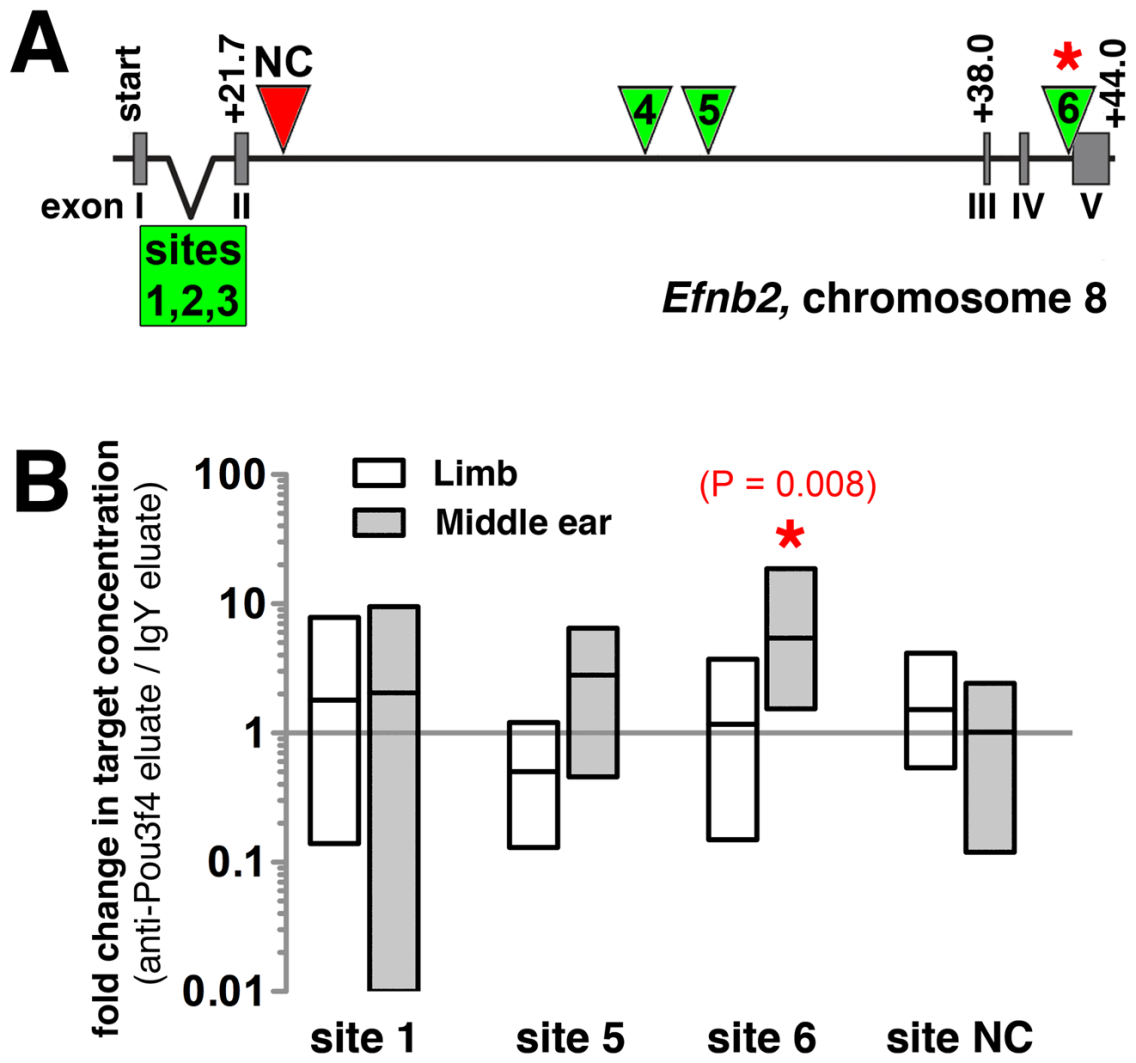
Enrichment of a putative Pou3f4 DNA binding site in anti-Pou3f4 middle ear ChIP eluate compared to IgY middle ear ChIP eluate. (**A**) Organization of the murine *Efnb2* genomic locus. Green triangles and box identify putative Pou3f4 DNA binding sites (ATTATTA motifs) at non-coding regions. Red triangle identifies the site of a selected negative control target lacking the ATTATTA motif. (**B**) Change in Pou3f4 DNA binding site abundance resulting from application of either anti-Pou3f4 and IgY IP, as assessed by qPCR and expressed in terms of fold changes for paired data (anti-Pou3f4 eluate/IgY eluate). Data for chromatin preparations from either E12.25 middle ear or E10.5 limb mesenchyme are shown. Each bar represents the range of fold change values obtained for sets of paired data (n = 8 Pou3f4 vs. IgY pulldown pairs, resulting from two qPCR trials with each of four independent chromatin preparations per tissue type; see Materials and Methods). Each horizontal line represents an average fold change.

To determine whether Pou3f4 physically interacts with the *Efnb2* locus in nascent middle ear mesenchyme, we first scanned the *Efnb2* genomic locus on mouse chromosome 8 for putative Pou3f4 DNA binding/regulatory motifs [37]. Six consensus motifs (ATTATTA) were distributed across intronic regions of *Efnb2* (Fig. 9A). Motifs at sites 3, 5, and 6 were fully conserved across two or more species (mouse/rat or mouse/rat/human), and the site 1 motif, though non-conserved, was localized to an intronic region of high phylogenetic conservation. We performed chromatin immunoprecipitation (ChIP) on sites 1, 3, 5, 6, as well as a non-consensus site (NC), using purified chicken anti-Pou3f4 IgY antibody and purified non-specific IgY as a negative control condition [28]. This was followed by SYBR-green-based qPCR and absolute quantification (see Materials and Methods) to measure concentrations of *Efnb2* target sequences in the anti-Pou3f4 and control IgY IPs. Chromatin was obtained from either E12.25 middle ear mesenchyme or E10.5 limb mesenchyme, the latter of which lacks detectable expression of Pou3f4 [31], [38]. Differences in target sequence concentration across paired experimental (anti-Pou3f4) and control (IgY) sample sets were assessed for each site/tissue combination.

Site 1, 3, 5, 6, and NC target concentrations in anti-Pou3f4 (experimental) and IgY (control) eluates were statistically indistinguishable using immunoprecipitated chromatin from limb mesenchyme (Fig. 9B; data not shown). Likewise, using immunoprecipitated chromatin from middle ear mesenchyme, site 1, 5, and NC target concentrations in anti-Pou3f4 and IgY eluates were statistically indistinguishable (Fig. 9B). Site 3 could not be evaluated statistically in middle ear chromatin eluates due to lack of fluorescence signal across 40 PCR cycles in roughly one-half of the assays. However, experimental and control eluates of middle ear chromatin differed significantly in their concentrations of target site 6 (p = 0.008; Wilcoxon signed rank test), with anti-Pou3f4 eluates showing a 5.5 fold average enrichment over IgY eluates (Fig. 9B). These results suggest that Pou3f4 transcription factor physically associates with specific non-coding sequence of the *Efnb2* locus during early stages of middle ear development.

## Discussion

### Pou3f4 and Efnb2 function within a common pathway to promote temporal bone development

Our results indicate that *Efnb2* lies downstream of *Pou3f4* in a genetic pathway governing embryonic-stage development of the mouse temporal bone. Previous work demonstrates that *Pou3f4* transcript and gene product are expressed in proximal branchial mesenchyme (where the stapes and styloid process will form) and in mesenchyme surrounding the nascent cochlea as early as E10.5 [31]. Although *Efnb2* is expressed in early migrating cranial neural crest cells [39], we could not - by mRNA hybridization - identify *Efnb2* transcript in mesenchyme surrounding the otic epithelium until after E11.5. We found qualitatively identical malformations of the stapes, styloid process, internal auditory canal, and cochlear capsule in both *Pou3f4* ^Cre/Y^ and (Sox9-IRES-Cre-mediated) *Efnb2* CKO perinates, and – between E12.5 and E14.5 - *Efnb2* and *Pou3f4* expression overlapped in mesenchyme that contributes to the formation of these structures. In mesenchyme surrounding the E13 stapes and cochlear rudiments, *Efnb2* expression was attenuated in the *Pou3f4* ^Cre/Y^ mutant compared to control, but *Pou3f4* expression appeared unaltered by conditional loss of *Efnb2*. Conditional loss of *Efnb2* (by Sox9-IRES-Cre) correlated with a 2-fold greater occurrence of full stapes-styloid process coalition than that of *Pou3f4* ^Cre/Y^ mutants; this is consistent with the finding that *Efnb2* signal is attenuated (rather than eliminated) in mesenchyme surrounding the *Pou3f4* ^Cre/Y^ stapes rudiment. Adult double mutants lacking both *Pou3f4* and *Efnb2* (*Pou3f4* ^Cre/Y^;*Efnb2* ^flox/null^) also displayed a 2-fold increased occurrence of stapes-facial canal bony coalition over adult *Pou3f4* ^Cre/Y^ single gene mutants. Thus, with respect to this phenotypic feature, the effect of losing both genes appears to be neither additive nor synergistic compared to loss of *Efnb2* alone.

We also provide evidence that, at the developing mouse middle ear, *Efnb2* transcription is potentiated by a physical interaction between Pou3f4 and the *Efnb2* locus. Pou3f4 protein is localized to nuclei of prospective middle ear mesenchyme cells by E11.5 [31]. Using chromatin isolated from E12.25 nascent middle ear mesenchyme, we found a 5.5 fold enrichment of specific intronic sequence near the 3′ end of *Efnb2* in anti-Pou3f4 eluate compared to IgY eluate. The relatively modest (though statistically significant) magnitude of fold increase may be due to the following factors. Our manual dissection undoubtedly captured a heterogeneous isolate, with Pou3f4^+^:Efnb2^+^ cells forming a small minority of cells from which chromatin was obtained. *Efnb2* signal in the region of interest (surrounding the stapes and within the domain of *Pou3f4*) was attenuated but not entirely eliminated in the *Pou3f4* ^Cre/Y^ mutant, indicating that Pou3f4 activity is not an absolute requirement for *Efnb2* expression at this site. Transcriptional regulation of *Efnb2* is complex, as evidenced by ChIP and transactivation analyses of the 5′ promoter region in cultured mouse arterial endothelial cells [40]. This study [40] revealed strong transactivation of *Efnb2* from the 5′ promoter region by a TALE homeodomain superfamily member, Meis1. In chicken embryos of equivalent maturity to E12.5–13.5 mouse, *Meis1* is expressed broadly in proximal 2^nd^ brachial arch mesenchyme encompassing the stapes rudiment and surrounding structures [41]. In E14.5 mouse, *Meis1* is strongly and widely expressed in mesenchyme surrounding the ossicles [42]. These considerations suggest a model wherein region-specific potentiation of *Efnb2* expression in periotic/branchial mesenchyme occurs through specific binding of Pou3f4 to 3′ intronic sequence.

We focused our ChIP and quantitative genetic analyses on the middle ear (stapes-styloid/facial canal region in particular) because the phenotype was efficiently scored by careful inspection of whole temporal bone preparations, and this allowed adequate sample sizes for statistical comparisons of multiple mutant genotypes. However, additional qualitative similarities between *Pou3f4* and *Efnb2* mutant phenotypes described here (e.g., thin cochlear capsule; dilated internal auditory canal) and the finding of attenuated *Efnb2* mRNA signal in cochlear subcapsular mesenchyme of *Pou3f4* ^Cre/Y^ mutants compared to that of control (Fig. S6) raise the question of whether Pou3f4-mediated *Efnb2* regulation has a more comprehensive role in temporal bone development. A previous study indicates that *Epha4*, encoding a cognate receptor for Efnb2, is a direct target of Pou3f4 transcription factor within cochlear sub-capsular mesenchyme [28]. This regulatory relationship is associated with mesenchyme-dependent fasciculation of spiral (VIIIth) ganglion axons. It is therefore reasonable to ask whether Pou3f4 directly coordinates temporal-spatial patterns of transcription for cognate Eph and ephrin genes, the products of which then mediate developmental events through ligand-receptor interaction. By RNA in situ hybridization, we were unable to detect altered expression of *Ephb2* or *Epha4* at the developing *Pou3f4* ^Cre/Y^ middle ear. More sensitive techniques for assessing change in Eph receptor transcript levels may be required in future studies, as we found overlapping expression of *Pou3f4* and *Ephb2* in mesenchyme dorsal to the stapes and multiple Pou3f4 DNA binding site motifs distributed across non-coding regions of the *Ephb2* gene (Coate and Raft, unpublished observations).

How the genetic regulatory mechanism proposed here affects cellular behavior during ear development remains to be determined. Genetic fate mapping in the mouse reveals that both the stapes crura/head and styloid process derive from 2^nd^ pharyngeal arch neural crest [4], [5]. Embryological studies of humans [43]–[45] and rodents [46], [47] indicate that discrete bony elements of the middle ear originate by splitting of one or more continuous mesenchymal condensations. We have shown that a continuous domain of mesenchymal *Sox9* gene expression encompasses primordia of the ossicles, styloid process, and otic capsule at stage E12.5. In *Pou3f4* ^Cre/Y^ mutant embryos, attenuated *Efnb2* mRNA signal correlated with failure to properly pattern or downregulate *Sox9* mRNA signal between the nascent stapes and styloid process rudiments. It is therefore possible that Eph-Efnb2 signaling promotes splitting of a continuous mesenchymal condensation into separate elements through effects on gene expression, cell adhesion and re-arrangement, or some combination of these processes. Our evidence to date suggests that the stapes-styloid phenotype of Sox9-IRES-Cre-mediated Efnb2 CKO embryos is caused by a loss of forward signaling through one or more cognate Eph receptors, as we failed to find this phenotype in perinatal mouse mutants lacking only the Efnb2 C-terminus.

### Reverse genetics offers insight into the etiology of DFNX2/DFN3 conductive hearing loss

Conductive hearing loss is typically due to inefficient transfer of sound energy through the middle ear. However, the precise anatomical bases for conductive hearing loss in humans with *POU3F4* mutations and DFNX2/DFN3 remain in question. Early reports of DFNX2/DFN3-type patients cite congenital fixation of the stapes footplate or absence of the annular ligament as causing conductive hearing loss [48], [49]. Other investigators attribute reduced (or absent) stapes mobility to increased perilymph fluid pressure within the bony capsule, which could result from a widened internal auditory canal and abnormal communication with the sub-dural space [6], [50], [51]. More recently, it has been proposed that the dilated internal auditory canal and other bony abnormalities characteristic of DFNX2/DFN3 dissipate acoustic energy at the level of the inner ear and amplify bone-conducted sounds [52], [53]. This so-called ‘third-window effect’ confounds interpretation of routine audiometric testing and causes ‘inner ear conductive hearing loss’, which can occur in the presence of a normally functioning middle ear [54].

We found the internal auditory canal of perinatal *Pou3f4* ^Cre/Y^ and *Efnb2* CKO mutants to be dilated and dysmorphic at the cartilaginous stage of temporal bone development, thus extending previous findings on the adult *Pou3f4* mutant temporal bone [14]. By contrast, we found no apparent malformation of the developing stapes footplate/oval window in either mutant at perinatal stages, suggesting that the footplate/oval window dysplasia of adult *Pou3f4* hemizygous null males arises during post-natal ossification or bone remodeling. The stapes footplate/oval window dysplasia observed in our sampling of adult *Pou3f4* ^Cre/Y^ and *Pou3f4* ^Cre/Y^;*Efnb2* ^flox/null^ temporal bones is consistent with previous observations of *Pou3f4* hemizygous null mice by Samadi et al. [55], but is inconsistent with reports of stapes footplate fixation in DFNX2/DFN3 patients [48], [49]. Rather than a footplate fixation, the *Pou3f4* ^Cre/Y^, *Pou3f4* ^Cre/Y^:*Efnb2* ^flox/null^, and Sox9-IRES-Cre-mediated *Efnb2* CKO stapes was immobilized by a bony and/or cartilaginous connection to the facial canal/styloid process. In humans, this so-called supra-structure fixation of the stapes dorsal crus and facial canal is rare and of unknown genetic etiology [56].

What might account for the apparent differences in stapes/oval window/facial canal morphology across mice and humans lacking *Pou3f4*/*POU3F4* activity? Variation in the developmental regulation of *Pou3f4*/*POU3F4* expression is one likely explanation. Breakpoint analyses of DFNX2/DFN3 microdeletions, cross-species non-coding sequence comparisons, and transgenic activities of putative cis regulatory sequences suggest that the temporal-spatial pattern of Pou3f4/POU3F4 expression during ear development is a composite of multiple 5′ cis-enhancer activities; phylogenetically conserved enhancers are distributed across a 1Mb region and respond differentially to major signaling pathways in model organism transgenic assays [12], [57]–[59]. It is also relevant that a supra-structure fixation of the stapes and styloid process/facial canal has not previously been identified in mice carrying other null alleles of *Pou3f4* [14], [15], [17], and the question of whether genetic background influences stapes morphology in mice lacking *Pou3f4* has been raised [15]. Here again, developmental gene expression requiring integration of many signals across a vast 5′ regulatory region may be susceptible to background-specific modifiers. Finally, DFNX2/DFN3 anatomy may be incompletely characterized, given the small number of cases that have been documented radiologically and lack of post-mortem temporal bone pathology studies.

Fixation of the stapes and styloid process/facial canal is found in other targeted mouse mutants. Mice heterozygous for the BMP antagonist *noggin* show genetic background-specific and incompletely penetrant coalition of the stapes and styloid process/facial canal without other apparent temporal bone defects [60]. By contrast, Wnt1-Cre-mediated inactivation of the endothelin-A receptor can result in coalition of the stapes and styloid process in the context of a severely dysmorphic middle ear and jaw [61]. The current model of early-stage jaw development places Endothelin-1 and BMP signaling upstream of several transcription factor families (Dlx, Msx, Hand) that pattern dorso-ventral axes of the 1^st^ and 2^nd^ pharyngeal arches [62]. Dlx transcription factors are required for proper expression of another POU family transcription factor gene, *Pou3f3*, in pharyngeal arch mesenchyme, and *Pou3f3* loss-of-function causes coalition of the stapes and styloid process [63]. Functional redundancy of *Pou3f3* and *Pou3f4* is another reasonable explanation for the lack of a reported stapes fixation in previous characterizations of *Pou3f4* mutants, and may also explain the variable expressivity/incomplete penetrance of stapes-styloid process coalition in the sample of *Pou3f4* ^Cre/Y^ mutants characterized here.

To date, no association exists between *EFNB2* mutations and a human disease or congenital defect. Complete loss of the human gene activity may cause early embryonic lethality due to failed remodeling of the primary embryonic vasculature into a system of arteries and veins, as is the case in the mouse [39], [64]. However, a growing list of roles for *Efnb2* in forming the murine auditory-vestibular system [24], [28], [35], [65], [66] raises the possibility that regulatory or hypomorphic mutations of *EFNB2* underlie functional variation in human hearing and balance.

## Materials and Methods

### Animals

*Pou3f4* mutant females of mixed (B6/129:Swiss Webster:CD1) genetic background, in which *Cre* is fused in frame with the *Pou3f4* start codon [28], were crossed to congenic C57BL/6 *Efnb2* ^LacZ/+^ null heterozygote males [63] (Jax stock 006039) to obtain F1 hybrids, which were maintained through 11 generations of intercrosses. Sperm was cryopreserved from F1:N10 males carrying both null alleles. A second *Efnb2* allele, with exon1 flanked by loxP sites [67] (Jax stock 006042), was maintained in the homozygous state on the C57BL/6 background as a separate colony. All *Pou3f4* mutant specimens (and controls) analyzed were male progeny of F1 Pou3f4-Cre^+^;*Efnb2* ^+/+^ or Pou3f4-Cre^+^;*Efnb2* ^LacZ/+^ hybrids crossed to congenic C57BL/6 *Efnb2* ^+/+^ or *Efnb2* ^flox/flox^ mice. Therefore all *Pou3f4* mutants (and controls) analyzed were F2 generation mixed:C57BL/6 hybrids. In some experiments, Pou3f4-Cre^+^ mice were bred to C57BL/6 ROSA-YFP reporter mice (Jax stock 006148). PCR assay of the *Smcx* and Smcy genes [68] was used to determine the sex of embryos. A separate congenic C57BL/6 breeding colony was used to generate Sox9-IRES-Cre^+^;*Efnb2* ^LacZ/+^ double heterozygous mice, which were mated with *Efnb2* ^flox/flox^ mice to obtain congenic C57BL/6 *Efnb2* CKO and control embryos, as previously described [24]. Fixed specimens of mixed 129/CD1 strain *Efnb2* C-terminal deletion homozygotes (ephrin-B2^LacZ^ mice in [33]–[35]) and wild-type littermate controls were kindly provided by Dr. Mark Henkemeyer (UT Southwestern Medical Center). All PCR genotyping of targeted alleles was conducted as previously described. CD1 embryos were used for studies of normal gene expression, histology, and chromatin isolation. All animal experiments were carried out in strict accordance with recommendations set forth in the Guide for the Care and Use of Laboratory Animals of the National Institutes of Health. All protocols were approved by the Investigational Animal Care and Use Committee of the National Institute on Deafness and Other Communication Disorders.

### Tissue fixation and preparation

Pregnant dams were euthanized with an anesthetic concentration of CO_2_ and cervical dislocation. For Alcian Blue/Nuclear Fast Red histology at mid-gestational stages (E12–E14.5) whole embryos were immersed in methacarn fixative (6∶3∶1 methanol:chloroform:glacial Acetic Acid) for 1 to 5 hours, trimmed, rinsed in methanol, cleared sequentially in methyl benzoate and xylene (1×30–60 minutes each), infiltrated with paraffin, and cut serially in the transverse plane of the embryo at 7 micron thickness on a Leica RM2145 microtome. For Alcian Blue/Nuclear Fast Red histology at perinatal stages, heads were split mid-sagittally, immersion-fixed overnight at 4 degrees Celsius in 4% paraformaldehyde in PBS (pH 7.4), cryoprotected in 30% sucrose/PBS, embedded in OCT compound (Tissue-Tek), and cut serially in the sagittal plane at 7 micron thickness on a Leica CM 3050 S cryostat. For Weigert's Resorcin Fuchin staining of the stapedio-vestibular joint, adult temporal bones were dissected and immersion-fixed overnight at 4 degrees Celsius in 4% paraformaldehyde in PBS, decalcified in 0.1M EDTA in PBS, washed, cleared sequentially in graded alcohols and xylene, infiltrated with paraffin, and cut serially in the longitudinal plane of the ear at 7 micron thickness on a Leica RM2145 microtome. For anti-GFP immunofluorescence and RNA in situ hybridization, mid-gestation embryos were immersion-fixed overnight at 4 degrees Celsius in 4% paraformaldehyde in PBS, cryoprotected in 30% sucrose/PBS, embedded in OCT medium, and cut serially in the transverse plane at 7 micron thickness on a Leica CM 3050 S cryostat. For whole mount Alcian Blue/Alizarin Red S staining of mid-sagitally split perinatal or adult heads, skin and brain were removed and specimens placed in 95% Ethanol for 3–5 days at room temperature.

### Tissue processing

For Alcian Blue/Nuclear Fast Red histology, hydrated sections were dipped in 3% acetic acid for 3 minutes, stained in 1% Alcian Blue 8GX (Sigma A3157) at pH 2.5, washed in running water for 10 minutes, counterstained with Nuclear Fast Red (Vector Laboratories H-3403) for 5 minutes, washed in tap water for 10 minutes, dehydrated, cleared, and mounted in Permount. Weigert's Resorcin Fuchsin (EMS 26370) staining was carried out according to manufacturer's instructions. Anti-GFP immunofluorescence on tissue sections was performed by standard methods using a FITC-conjugated goat anti GFP antibody (Gene-Tex 1∶200), followed by counterstaining with rhodamine-phalloidin (Molecular Probes) and DAPI. RNA hybridzation to tissue sections was performed in slide mailers using digoxygenin-labeled probes in weakly acidic hybridization buffer (pH 4.5), anti-digoxigenin-AP Fab fragments (Roche 11093274910) in TBST buffer, and the NBT/BCIP colorimetric substrate reaction in AP buffer at pH 9.5. Whole mount Alcian Blue/Alizarin Red S staining of cartilage and bone was performed according to the method of McLeod [69].

*RNA probes.* Cre-mediated recombination at the floxed *Efnb2* allele was validated with a 140bp exon 1-specific fragment (NM_010111.5, nt 139-278) PCR cloned from C57BL/6 tail DNA and ligated into pCR4-TOPO vector for in vitro transcription. Wild-type *Efnb2* was detected with a ∼1 kb probe transcribed from NM_010111.5, nt136-1188. Other cDNAs used were as follows: *Pou3f4* (NM_008901, a 511 base-pair NcoI fragment), *Sox9* (NM-011448, 500 base-pair fragment), *Epha4* (NM_007936, nt2782-4242), *Ephb2* (NM_010142), *Ephb3* (NM_010143, nt535-1207), and *Ephb4* (BC090839.1, nt988-1947).

### Histological and Anatomical Analyses

For qualitative comparisons of hybridization signals across genotypes, mutant-control littermate pairs were serially sectioned on the same day; each slide for a littermate pair contained mutant and control sections and these were distributed in equal proportions across the minimum number of slides needed to uniformly sample entire domains of interest from every second 7-micron serial section. Mutant and control sections were randomized in their placement from slide to slide. Four separate hybridization experiments were conducted, each containing one or more littermate pair section sets probed for each of the following genes: *Efnb2*, *Ephb2*, ***Epha4*** **,** ***Pou3f4*** **, and** ***Sox9***.

Digital images of all sections in the area of the stapes were uniformly captured for each littermate pair/probe set during a single imaging session, and extent and intensity of signal in the area of the mutant and control stapes was rated from raw TIFF images. Scoring of defects at the stapes-styloid process or stapes-facial canal in perinatal or adult ears was accomplished by partial dissection and observation under a stereo-dissecting microscope and digital capture of images on a Leica M205FA stereomicroscope with motorized focusing. Statistical analyses of categorical data (using Prism 5 software) were carried out using a two-sided Fisher's exact test for 2×2 contingencies or a Chi Square test for data sets with 2 or more degrees of freedom. In the text, ‘occurrence’ denotes outcomes where *n* =  the number of temporal bones analyzed; ‘penetrance’ denotes outcomes where *n* =  the number of animals analyzed.

### Chromatin Immunoprecipitation (ChIP)-PCR

A sharpened stainless-steel wire was used to isolate blocks of nascent middle ear mesenchyme from stage E12.25 CD1 mouse embryos. We generated four independent biological samples, each of which contained pooled tissue from eight ears. Each independent sample was collected in ice-cold 1X PBS, fixed in 4% paraformaldehyde for 20 minutes at room temperature, washed, pelleted, and frozen. Chromatin was isolated from each pellet by methods outlined in the Pierce Agarose ChIP Kit (Thermo Scientific, Catalog #26156). Chromatin was treated with micrococcal nuclease for 15 minutes at 37°C to produce DNA fragments ranging in size from 200–600 base pairs. Chromatin from E10.5 limb mesenchyme (where Pou3f4 is not expressed) [31], [38] was identically prepared. Each independent chromatin preparation was divided equally by volume and incubated overnight at 4 degrees Celcius with either 10 µg whole IgY (Jackson Immunoresearch, Catalog #003-000-003) or 10 µg anti Pou3f4-specific IgY [28]. Following incubation, Preciphen beads (Aves Labs, Catalog #P-1010) were added to the antibody/chromatin mixture and allowed to bind for 2 hours. After extensive rinsing in low- and high-salt buffers, antibody/chromatin complexes were eluted from the beads, treated with proteinase K, and column purified.

Concentrations of *Efnb2* target sequences in IP eluates were quantified by qPCR on an Applied Biosystems StepOne Realtime PCR system using SYBR-green and the following primer sets derived from mouse chromosome 8 sequence GRCm38.p2 C57BL/6J (NC_000074.6): a negative control site lacking the Pou3f4 binding motif (non-consensus or NC site) CTTGTTCCCAGTGTGGATGA (forward) and ACCCCAAACAACTGAACCAG (reverse); site 1, TTACGAATTGGACACTAACAAGC (forward) and TGGCCTGAAAAACAGGTTC (reverse); site 3, ACACTAACAAGCCTCTTCTCCA (forward) and TGCAGGAATATAAGTGGCCTGA (reverse); site 5, TTTGGCTTTTCCTGGACATT (forward) and GCCCAAGTTAATGCGTTTTC (reverse); site 6, GACCTTGAGGCTCCTTTGC (forward) and GCAGAAACCCCGAAATGTAA (reverse). The annealing temperature used for all reactions was 55°C, with the exception of the site 3 primer pair reactions, which used an annealing temperature of 60°C. All qPCR reactions comprised 40 cycles and resulted in a single product, as determined by melt curve and gel electrophoretic analyses. Amplicon specificity was validated by direct sequencing. For absolute quantifications of template input to qPCR reactions, a standard curve was generated for each primer pair using total genomic DNA (gDNA) from pooled forelimb and hindlimb tissues of E10.5 CD1 mouse embryos; gDNA was purified, fixed, nuclease-digested as described above, and serially diluted for use in triplicate qPCR reactions. Semi-log transformation and linear regression gave Pearson correlation coefficients ranging from −.963 to −.999. Primer efficiencies, defined by the equation 10∧[−1/slope], ranged from 1.9 to 2.7. Concentrations of target site DNA from IgY and anti-Pou3f4 pull-downs were computed from Ct values and standard curve equations. Each of four independent chromatin preparations (middle ear or limb) was assayed twice by qPCR in technical triplicate, yielding 8 matched pairs of data (anti-Pou3f4 IP vs. control IgY IP) for each target site tested. Paired data sets were subjected to the Wilcoxon signed rank test (Prism 5 software), with a cut-off for statistical significance at P<0.05.

## Supporting Information

Figure S1 **Dilation and incomplete septation of the internal auditory canal in** ***Pou3f4*** **^Cre/Y^,** ***Pou3f4*** **^Cre/Y^;** ***Efnb2*** **^flox/null^, and Sox9-IRES-Cre^+^;** ***Efnb2*** **^flox/null^ mutants.** (**A–C**) Medial views of wild-type and mutant temporal bones excised from 16–20 week old adult mice and stained with alizarin red/alcian blue, shown to scale. Bar  = 100 microns. Foramina of the internal auditory canal are highlighted by dotted lines. Three foramina are evident in wild-type (A); there is no septation of foramina ii (for superior vestibular VIIIth nerve) and iii (for VIIth nerve) in the mutants (B,C). Foramen i conducts the auditory VIIIth nerve branch). (**D–F**) Medial views of wild-type and mutant cartilaginous capsules from E19-P0 heads stained with alizarin red/alcian blue, shown to scale. Bar  = 100 microns. Three foramina are evident in wild-type; arrow in (A) highlights septation of foramina ii and iii. In mutants (E, F), the internal auditory canal is enlarged compared to wild-type and there is no septation of foramina ii and iii. Asterisk in (E) highlights cartilage outside the focal plane of the capsule medial wall. Axes in A apply to all photos. R =  rostral.
(TIF)

Figure S2 **Facial canal lateral wall is formed by fusion and growth of the endochondral styloid process and intramembranous tympanic ring.** (**A–C**) Lateral views of excised, Alcian Blue/Alizarin Red-stained temporal bones from wild-type mice at post-natal days 5, 8, and 15. Grey dots highlight the tympanic ring dorsal edge at its apposition with the styloid process (sty). The endochondral styloid process is cartilaginous and stains blue at P5 and P8; the intramembranous tympanic ring (tr), is ossified at birth and stains red at all stages shown. s, stapes; i, incus; m, malleus. (**D–F**) Magnified views of the boxed regions in (A–C), respectively. Arrows indicate apparent vectors of tympanic ring growth; the tympanic ring is superficial to the styloid process and its expansion fully obscures the styloid process by P15. (**G–I**) show ventral views of the specimens shown in (A–C), respectively, with tympanic ring (tr) and malleus (m) dissected away for unobscured views of the stapes (s), facial canal (dotted line), and styloid process (sty). Note the near-complete ossification of the styloid process between stages P8 and P15.
(TIF)

Figure S3 **Validation of Sox9-IRES-Cre-mediated recombination at the** ***Efnb2*** **locus.** Sections of control (A) and *Efnb2* CKO littermates (B), showing the middle ear at stage E14.5, hybridized with an *Efnb2* exon1-specific probe. Signal from the *Efnb2* exon1 probe in developing ear and second branchial arch tissues is markedly decreased in the mutant compared to control. asterisk, VIIth nerve; s, stapes; m, malleus; c, otic capsule; cd, cochlear duct; spg, spiral ganglion.
(TIF)

Figure S4 **Homozygous deletion of the Efnb2 C-terminus has no apparent effect on stapes and styloid process morphology.** Sagittal sections of mixed 129/CD1 strain wild-type control (A) and *Efnb2* ^C-del/C-del^ (B) littermates at stage E18.5, stained with Toluidine Blue to reveal cartilage. Distance between the stapes (sta) and styloid process (sty) is similar across genotypes. Scale bar  = 100 micrometers.
(TIF)

Figure S5 ***Efnb2*** **mRNA signal intensity is attenuated relative to control in** ***Pou3f4*** **^Cre/Y^ mutant mesenchyme dorsal to the stapes at stages E13–13.5.** Representative image data for 5 of 6 wild-type (A–E) and mutant (A′–E′) littermate pairs, hybridized under controlled conditions to assay for potential change in *Efnb2* expression across genotypes. Brackets in (A, A′) highlight mesenchyme dorsal to the stapes (s) and surrounding the VIIth nerve (asterisk), where *Efnb2* hybridization signal is specifically altered across genotypes. Structures are identically framed in all panels. *Efnb2* signals at the malleus (m) and otic epithelium (oe) appear similar across genotypes. Scale bar  = 100 micrometers. The fifth of six wild-type/mutant pairs analyzed is shown in the main body of the text (Fig. 8A, A′, B, B′).
(TIF)

Figure S6 ***Efnb2*** **mRNA signal intensity is attenuated relative to control in** ***Pou3f4*** **^Cre/Y^ mutant sub-capsular mesenchyme at stage E13.** Transverse sections of control (A) and *Pou3f4* ^Cre/Y^ (B) E13.0 littermates hybridized to detect *Efnb2*. Red arrowheads highlight attenuation of *Efnb2* hybridization signal in mutant sub-capsular mesenchyme surrounding the cochlea and spiral ganglion. Spiral ganglia are bounded by black dotted lines; cochleae are encircled by solid white lines. Scale bar  = 100 micrometers.
(TIF)

Figure S7 ***Sox9*** **marks a continuous domain comprising branchial arch cartilages, ossicles, styloid process, and otic capsule at stage E12.5.** (**A–F**) Selected images from serial transverse sections through the 1^st^ and 2^nd^ branchial arches of an E12.5 embryo that were hybridized to detect *Sox9* mRNA. Images are arranged in an anterior to posterior sequence (A through F). Note that *Sox9* expression bridges all otic and branchial arch rudiments specified. Meckel's rudiment (Me), a dark-staining bar in (A) is cartilaginous at this stage; all other rudiments are mesenchymal condensations. Blue arrowheads in (C,D) highlight *Sox9* signal bridging the stapes (s) and styloid process (sty). Asterisks in (C,D) highlight the VIIth cranial nerve. m/i, malleus/incus condensation; oc, otic capsule condensation; m, caudal end of the malleus/incus condensation in the 2^nd^ arch; R, Reichert's cartilage rudiment, ie, inner ear; hb, hindbrain; ph, pharynx.
(TIF)

Table S1 **Absolute frequency and prevalence values for all possible phenotypic categories of stapes-styloid/facial canal connection in** ***Pou3f4*** **^Cre/Y^ and** ***Efnb2*** **mutant samples.** Sample number (*n*) refers to the number of mice analyzed.
(PDF)

## References

[1] Adams JC, Liberman MC (2010) Anatomy. In: Merchant SN Nadol Jr JB, editors. Schuknecht's Pathology of the Ear. 3rd ed. Shelton: People's Medical Publishing House-USA. pp. 54–95.

[2] Depew MJ, Tucker AS, Sharpe PT (2002) Craniofacial Development. In: Rossant J, Tam PPL, editors. Mouse Development: Patterning, Morphogenesis, and Organogenesis. Academic Press. pp. 421–481.

[3] McBratney-Owen B, Iseki S, Bamforth SD, Olsen BR, Morriss-Kay GM (2008) Development and tissue origins of the mammalian cranial base. Dev Biol 322: 121–132. 18680740 10.1016/j.ydbio.2008.07.016 PMC2847450

[4] O'Gorman S (2005) Second branchial arch lineages of the middle ear of wild-type and Hoxa2 mutant mice. Dev Dyn 234: 124–131. 15861402 10.1002/dvdy.20402

[5] Thompson H, Ohazama A, Sharpe PT, Tucker AS (2012) The origin of the stapes and relationship to the otic capsule and oval window. Dev Dyn 241: 1396–1404. 22778034 10.1002/dvdy.23831

[6] Cremers CW, Hombergen GC, Scaf JJ, Huygen PL, Volkers WS, et al (1985) X-linked progressive mixed deafness with perilyphatic gusher during stapes surgery. Arch Otolarygol 111(4): 249–254. 10.1001/archotol.1985.00800060073010 3977755

[7] Phelps PD, Reardon W, Pembrey M, Bellman S, Luxom L (1991) X-linked deafness, stapes gushers and a distinctive defect of the inner ear. Neuroradiology 33: 326–330. 1922747 10.1007/BF00587816

[8] Bach I, Brunner HG, Beighton P, Ruvalcaba RHA, Reardon W, et al (1992) Microdeletions in patients with gushjer-associated X-linked mixed deafness (DFN3). Am J Hum Genet 51: 38–44. 1609803 PMC1682865

[9] Piussan C, Hanauer A, Dahl N, Mathieu M, Kolski C, et al (1995) X-linked progressive mixed deafness: A new microdeletion that involves a more proximal region in Xq21. Am J Hum Genet 56: 224–230. 7825582 PMC1801308

[10] Huber I, Bitner-Glindzicz M, de Kok YJ, van der Maarel SM, Ishikawa-Brush Y, et al (1994) X-linked mixed deafnes (DFN3): cloning and characterization of the critical region allows the identification of novel microdeletions. Hum Mol Genet 3(7): 1151–1154. 7981685 10.1093/hmg/3.7.1151

[11] de Kok YJ, van der Maarel SM, Bitner-Glindzicz M, Huber I, Monaco AP, et al (1995) Association between X-linked mixed deafness and mutations in the POU domain gene POU3F4. Science 267: 685–688. 7839145 10.1126/science.7839145

[12] de Kok YJ, Vossenaar ER, Cremers CW, Dahl N, Laporte J, et al (1996) Identification of a hot spot for microdeletions in patients with X-linked deafness type 3 (DFN3) 900 kb proximal to the DFN gene POU3F4. Hum Mol Genet 5(9): 1229–1235. 8872461 10.1093/hmg/5.9.1229

[13] Ryan AK, Rosenfeld MG (1997) POU domain family values: flexibility, partnerships, and developmental codes. Genes Dev 11(10): 1207–1225. 9171367 10.1101/gad.11.10.1207

[14] Phippard D, Lu L, Lee D, Saunders JC, Crenshaw EB 3rd (1999) Targeted mutagenesis of the POU-domain gene Brn4/Pou3f4 causes developmental defects in the inner ear. J Neurosci 19(14): 5980–5989. 10407036 10.1523/JNEUROSCI.19-14-05980.1999 PMC6783103

[15] Phippard D, Boyd Y, Reed V, Gisher G, Masson WK, et al (2000) The sex-linked fidget mutation abolishes Brn4/Pou3f4 gene expression in the embryonic inner ear. Hum Mol Genet 9(1): 79–85. 10587581 10.1093/hmg/9.1.79

[16] Braunstein EM, Crenshaw EB 3rd, Morrow BE, Adams JC (2000) Cooperative function of Tbx1 and Brn4 in the periotic mesenchyme is necessary for cochlea formation. J Assoc Res Otolarygol 9(1): 33–43. 10.1007/s10162-008-0110-6 PMC2536808 18231833

[17] Minowa O, Ikeda K, Sugitani Y, Oshima T, Nakai S, et al (1999) Altered cochlear fibrocytes in a mouse model of DFN3 non-syndromic deafness. Science 285: 1408–1411. 10464101 10.1126/science.285.5432.1408

[18] Song MH, Choi S-Y, Wu L, Oh S-K, Lee HK, et al (2011) Pou3f4 deficiency causes defects in otic fibrocytes and stria vascularis by different mechanisms. Biochem Biophys Res Comm 404: 528–533. 21144821 10.1016/j.bbrc.2010.12.019

[19] Cowan CA, Henkemeyer M (2002) Ephrins in reverse, park and drive. Trends Cell Biol 12: 339–346. 12185851 10.1016/s0962-8924(02)02317-6

[20] Xu NJ, Henkemeyer M (2012) Ephrin reverse signaling in axon guidance and synaptogenesis. Semin Cell Dev Biol 23: 58–64. 22044884 10.1016/j.semcdb.2011.10.024 PMC3288821

[21] Klein R (2012) Eph/ephrin signaling during development. Development 139: 4105–4109. 23093422 10.1242/dev.074997

[22] Batlle E, Wilkinson DG (2012) Molecular mechanisms of cell segregation and boundary formation in development and tumorigenesis. Cold Spring Harb Perspect Biol 4: a008227. 22214769 10.1101/cshperspect.a008227 PMC3249626

[23] Bush JO, Soriano P (2010) Ephrin-B1 forward signaling regulates craniofacial morphogenesis by controlling cell proliferation across Eph-ephrin boundaries. Genes Dev 24: 2068–2080. 20844017 10.1101/gad.1963210 PMC2939368

[24] Raft S, Andrade LR, Shao D, Akiyama H, Henkemeyer M, et al (2014) Ephrin-B2 governs morphogenesis of endolymphatic sac and duct epithelia in the mouse inner ear. Dev Biol 390(1): 51–67. 24583262 10.1016/j.ydbio.2014.02.019 PMC4113727

[25] Holmberg J, Genander M, Halford MM, Anneren C, Sondell M, et al (2006) EphB receptors coordinate migration and proliferation in the intestinal stem cell niche. Cell 125: 1151–1163. 16777604 10.1016/j.cell.2006.04.030

[26] Genander M, Halford MM, Xu N-J, Eriksson M, Yu Z (2009) Dissociation of EphB2 signaling pathways mediating progenitor cell proliferation and tumor suppression. Cell 139: 679–692. 19914164 10.1016/j.cell.2009.08.048 PMC2786256

[27] Chumley MJ, Catchpole T, Silvany RE, Kernie SG, Henkemeyer M (2007) EphB receptors regulate stem/projenitor cell proliferation, migration, and polarity during hippocampal neurogenesis. J Neurosci 27(49): 13481–13490. 18057206 10.1523/JNEUROSCI.4158-07.2007 PMC6673089

[28] Coate TM, Raft S, Zhao X, Ryan AK, Crenshaw EB 3rd, et al (2012) Otic mesenchyme cells regulate spiral ganglion axon fasciculation through a Pou3f4/EphA4 signaling pathway. Neuron 73(1): 49–63. 22243746 10.1016/j.neuron.2011.10.029 PMC3259535

[29] Ohashi M, Ide S, Kimitsuki T, Komune S, Suganuma T (2006) Three dimensional regular arrangement of the annular ligament of the rat stapediovestibular joint. Hear Res 213: 11–16. 16476532 10.1016/j.heares.2005.11.007

[30] Sobol SE, Teng X, Crenshaw EB 3rd (2005) Abnormal mesenchymal differentiation in the suprerior semicircular canal of Brn4/Pou3f4 knockout mice. Arch Otolarygol Head Neck Surg 131: 41–45. 10.1001/archotol.131.1.41 15655183

[31] Phippard D, Heydemann A, Lechner M, Lu L, Lee D, et al (1998) Changes in subcellular localization of the Brn4 gene product precede mesenchymal remodeling of the otic capsule. Hear Res 120: 77–85. 9667433 10.1016/s0378-5955(98)00059-8

[32] McPhee JR, Van De Water TR (1985) A comparison of morphological stages and sulfated glycosaminoglycan production during otic capsule formation: in vivo and in vitro. Anat Rec 213(4): 566–577. 4083537 10.1002/ar.1092130413

[33] Cowan CA, Yokoyama N, Saxena A, Chumley MJ, Silvany RE, et al (2004) Ephrin-B2 reverse signaling is required for axon pathfinding and cardiac valve formation but not early vascular development. Dev Biol 271: 263–271. 15223333 10.1016/j.ydbio.2004.03.026

[34] Dravis C, Yokoyama N, Chumley MJ, Cowan CA, Silvany RE, et al (2004) Bidirectional signaling mediated by ephrin-B2 and EphB2 controls urorectal development. Dev Biol 271: 272–290. 15223334 10.1016/j.ydbio.2004.03.027

[35] Dravis C, Wu T, Chumley MJ, Yokoyama N, Wei S, et al (2007) EphB2 and ephrin-B2 regulate the ionic homeostasis of vestibular endolymph. Hear Res 223: 93–104. 17158005 10.1016/j.heares.2006.10.007

[36] Akiyama H, Kim J-E, Nakashima K, Balmes G, Iwai N, et al (2005) Osteo-chondroprogenitor cells are derived from Sox9 expressing precursors. Proc Natl Acad Sci U S A 102: 14665–14670. 16203988 10.1073/pnas.0504750102 PMC1239942

[37] Okazawa H., Imafuku I., Minowa M.T., Kanazawa I., Hamada H., et al (1996) Regulation of striatal D1A dopamine receptor gene transcription by Brn-4. Proc. Natl. Acad. Sci. USA 93, 11933–11938. 8876240 10.1073/pnas.93.21.11933 PMC38161

[38] Heydemann A, Nguyen LC, Crenshaw EB 3rd (2001) Regulatory regions from the Brn4 promoter direct LACZ expression to the developing forebrain and neural tube. Brain research Developmental brain research 128: 83–90. 11356266 10.1016/s0165-3806(01)00137-7

[39] Adams RH, Diella F, Hennig S, Helmbacher F, Deutsch U, et al (2001) The cytoplasmic domain of the ligand ephrinB2 is required for vascular morphogenesis but not neural crest migration. Cell 104, 57–69. 11163240 10.1016/s0092-8674(01)00191-x

[40] Sohl M, Lanner F, Farnebo F (2009) Characterization of the murine Ephrin-B2 promoter. Gene 437(1–2): 54–59. 19268698 10.1016/j.gene.2009.02.017

[41] Sanchez-Guardado LO, Ferran JL, Rodriguez-Gallardo L, Puelles L, Hidalgo-Sanchez M (2011) Meis gene expression patterns in the developing chicken inner ear. J Comp Neurol 519: 125–147. 21120931 10.1002/cne.22508

[42] Diez-Roux G, Banfi S, Sultan M, Geffers L, Anand S, et al (2011) A high-resolution anatomical atlas of the transcriptome in the mouse embryo. Plos Biol 9(1): e10000582. 10.1371/journal.pbio.1000582 PMC3022534 21267068

[43] Hanson JR, Anson BJ, Strickland EM (1962) Branchial sources of the auditory ossicles in man. Part II: Observations of embryonic stages from 7mm to 28 mm (CR length). Arch Otolaryng 76: 200–215. 13904456 10.1001/archotol.1962.00740050208004

[44] Cauldwell EW, Anson BJ (1942) Stapes, fissula ante fenestram, and associated structures in man. Arch Otolaryngol 36: 891–925. 10.1001/archotol.1948.00690040274001 18118915

[45] Rodriguez-Vasquez JF (2005) Development of the stapes and associated structures in human embryos. J Anat 207: 165–173. 16050903 10.1111/j.1469-7580.2005.00441.x PMC1571512

[46] Amin S, Matalova E, Simpson C, Yoshida H, Tucker AS (2007) Incudomalleal joint formation: the roles of apoptosis, migration, and downregulation. BMC Dev Biol 7: 134. 18053235 10.1186/1471-213X-7-134 PMC2222641

[47] Amin S, Tucker AS (2006) Joint formation in the middle ear: lessons from the mouse and guinea pig. Dev Dyn 235: 1326–1333. 16425222 10.1002/dvdy.20666

[48] Nance WE, Steleff R, McLeod A, Sweeney A, Cooper C, et al (1971) X-linked mixed deafness with congenital fixation of the stapedial footplate and perilymphatic gusher. Birth Defects Orig Art Ser VII(4): 64–69. 5173351

[49] Glasscock ME (1973) The stapes gusher. Arch Otolaryngol 98: 82–91. 4723769 10.1001/archotol.1973.00780020088004

[50] Cremers CW, Hombergen GC, Wentges RT (1983) Perilymphatic gusher and stapes surgery. A predictable complication? Clin Otolaryngol Allied Sci 8(4): 235–240. 6652936 10.1111/j.1365-2273.1983.tb01434.x

[51] Cremers CW (1985) Audiologic features of the X-linked progressive mixed deafness syndrome with perilymphatic gusher during stapes surgery. Am J Otol 6(3): 243–246. 4039896

[52] Snick A, Hombergen G, Mylanus E, Cremers CW (1995) Air-bone gap in patients with X-linked stapes gusher syndrome. Am J Otol 16(2): 241–246. 8572127

[53] Choi BY, An Y-H, Park JH, Jang JH, Chung HC, et al (2013) Audiological and surgical evidence for the presence of a third window effect for the conductive hearing loss in DFNX2 deafness irrespective of type of mutations. Eur Arch Otorhinolaryngol 270: 3057–3062. 23400403 10.1007/s00405-013-2386-3

[54] Merchant SN, Rosowski JJ (2008) Conductive hearing loss caused by third-window lesions of the inner ear. Otol Neurotol 29: 282–289. 18223508 10.1097/mao.0b013e318161ab24 PMC2577191

[55] Samadi DS, Saunders JC, Crenshaw EB 3rd (2005) Mutation of the POU-domain gene Brn4/Pou3f4 affects middle ear sound conduction in the mouse. Hear Res 199: 11–21. 15574296 10.1016/j.heares.2004.07.013

[56] Nandapalan V, Tos M (2000) Isolated congenital stapes ankylosis: an embryologic survey and literature review. Am J Otol 21: 71–80. 10651438

[57] Ahn KJ, Passero F, Crenshaw EB 3rd (2009) Otic mesenchyme expression of cre recombinase directed by the inner ear enhancer of the Brn4/Pou3f4 gene. Genesis 47: 137–141. 19217071 10.1002/dvg.20454

[58] Naranjo S, Voesenek K, de la Calle-Mustienes E, Robert-Moreno A, Kokotas H (2010) Multiple enhancers located in a 1-Mb region upstream of POU3F4 promote expression during inner ear development and may be required for hearing. Hum Genet 128: 411–419. 20668882 10.1007/s00439-010-0864-x PMC2939330

[59] Robert-Moreno A, Naranjo S, de la Calle-Mustienes E, Gomez-Karmeta JL, Alsina B (2010) Characterization of new otic enhancers of the Pou3f4 gene reveal distinct signaling pathway regulation and spatio-temporal patterns. PLoS One 5(12): e15907. 21209840 10.1371/journal.pone.0015907 PMC3013142

[60] Hwang C-H, Wu DK (2008) Noggin heterozygous mice: an animal model for congenital conductive hearing loss in humans. Hum Mol Genet 17(6): 844–853. 18096605 10.1093/hmg/ddm356

[61] Ruest L-B, Clouthier DE (2009) Elucidating timing and function of endothelin-A receptor signaling during craniofacial development using neural crest cell-specific gene deletion and receptor anatagonsim. Dev Biol 328: 94–108. 19185569 10.1016/j.ydbio.2009.01.005 PMC2821796

[62] Medeiros DM, Crump JG (2008) New perspectives on pharyngeal dorsoventral patterning in development and evolution of the vertebrate jaw. Dev Biol 371: 121–135. 10.1016/j.ydbio.2012.08.026 PMC3466404 22960284

[63] Jeong J, Li X, McEvilly RJ, Rosenfeld MG, Lufkin T, et al (2008) Dlx genes pattern mammalian jaw primordium by regulating both lower jaw-specific and upper jaw-specific genetic programs. Development 135: 2905–2916. 18697905 10.1242/dev.019778 PMC4913551

[64] Wang HU, Chen ZF, Anderson DJ (1998) Molecular distinction and angiogenic interaction between embryonic arteries and veins revealed by ephrin-B2 and its receptor Eph-B4. Cell 93: 741–753. 9630219 10.1016/s0092-8674(00)81436-1

[65] Miko IJ, Henkemeyer M, Cramer KS (2008) Auditory brainstem responses are impaired in EphA4 and ephrin-B2 deficient mice. Hear Res 235: 39–46. 17967521 10.1016/j.heares.2007.09.003 PMC3250224

[66] Zhou CQ, Lee J, Henkemeyer MJ, Lee KH (2011) Disruption of EphrinB/Eph B interaction results in abnormal cochlear innervation patterns. Laryngoscope 121: 1541–1547. 21647913 10.1002/lary.21861

[67] Gerety SS, Anderson DJ (2002) Cardiovascular ephrinB2 function is essential for embryonic angiogenesis. Development 129: 1397–1410. 11880349 10.1242/dev.129.6.1397

[68] Mroz K, Carrel L, Hunt PA (1999) Germ cell development in the XXY mouse: Evidence that X chromosome reactivation is independent of sexual differentiation. Dev Biol 207(1): 229–238. 10049577 10.1006/dbio.1998.9160

[69] McLeod MJ (1980) Differential staining of cartilage and bone in whole mouse fetuses by Alcian Blue and Alizarin Red S. Teratology 22: 299–301. 6165088 10.1002/tera.1420220306

