# Supplementary figures and images for: Pou3f4-Mediated Regulation of Ephrin-B2 Controls Temporal Bone Development in the Mouse
Source: PLoS One. 2014 Oct 9;9(10):e109043. doi: 10.1371/journal.pone.0109043 (PMC4192298; doi:10.1371/journal.pone.0109043)

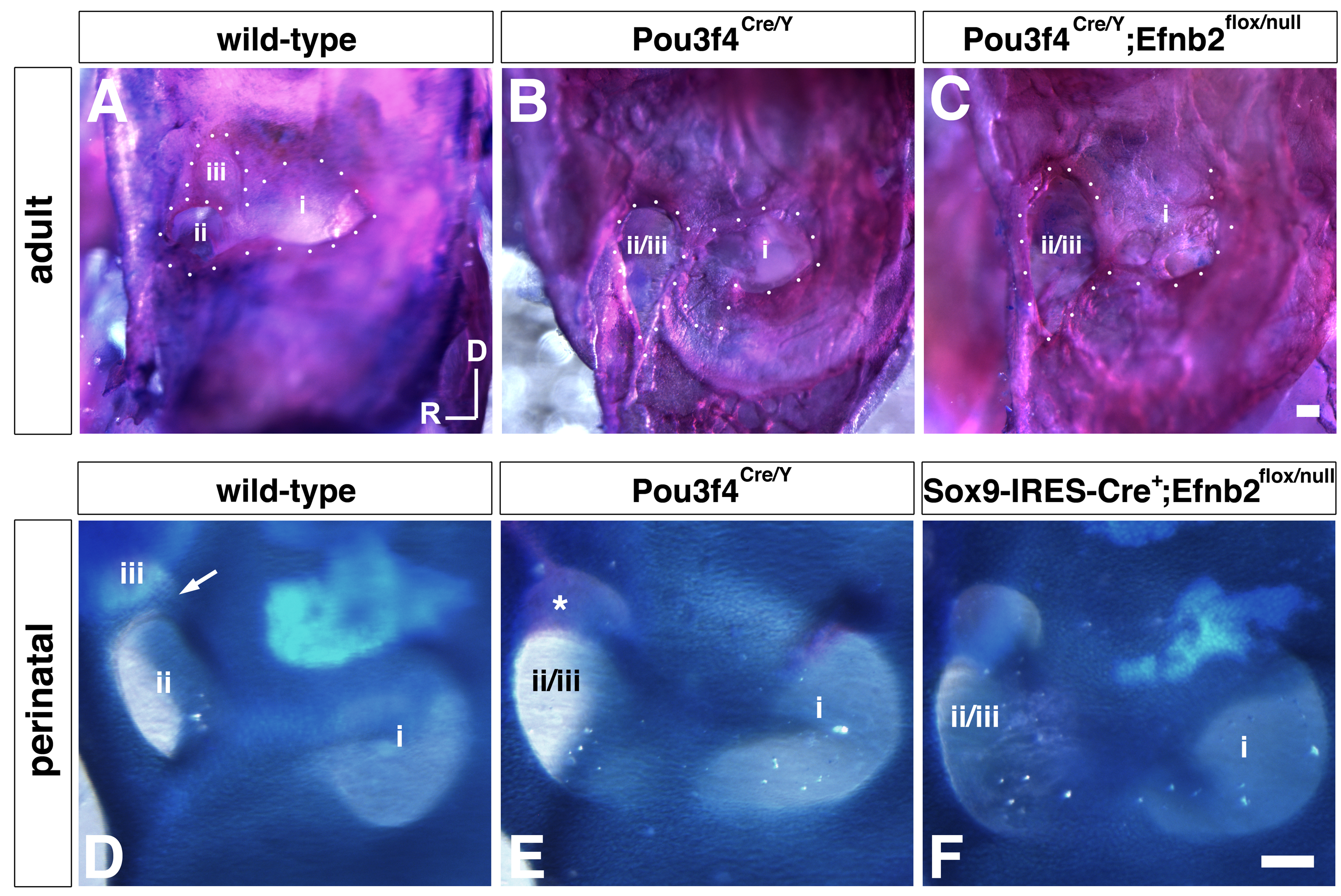

Supplement: Figure S1 — Dilation and incomplete septation of the internal auditory canal in Pou3f4 Cre/Y, Pou3f4 Cre/Y; Efnb2 flox/null, and Sox9-IRES-Cre+; Efnb2 flox/null mutants. (A–C) Medial views of wild-type and mutant temporal bones excised from 16–20 week old adult mice and stained with alizarin red/alcian blue, shown to scale. Bar = 100 microns. Foramina of the internal auditory canal are highlighted by dotted lines. Three foramina are evident in wild-type (A); there is no septation of foramina ii (for superior vestibular VIIIth nerve) and iii (for VIIth nerve) in the mutants (B,C). Foramen i conducts the auditory VIIIth nerve branch). (D–F) Medial views of wild-type and mutant cartilaginous capsules from E19-P0 heads stained with alizarin red/alcian blue, shown to scale. Bar = 100 microns. Three foramina are evident in wild-type; arrow in (A) highlights septation of foramina ii and iii. In mutants (E, F), the internal auditory canal is enlarged compared to wild-type and there is no septation of foramina ii and iii. Asterisk in (E) highlights cartilage outside the focal plane of the capsule medial wall. Axes in A apply to all photos. R = rostral. (TIF) [file pone.0109043.s001.tif]

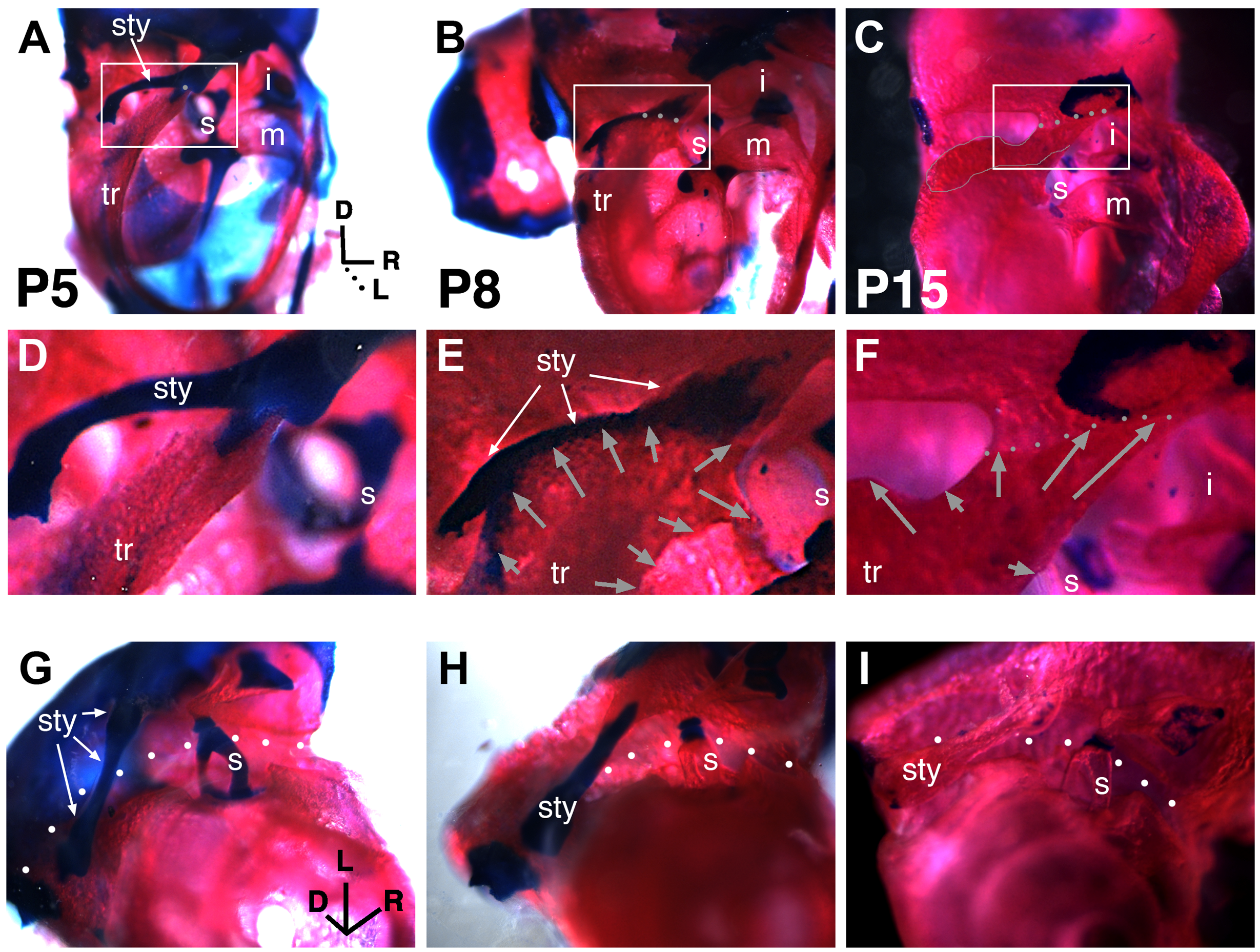

Supplement: Figure S2 — Facial canal lateral wall is formed by fusion and growth of the endochondral styloid process and intramembranous tympanic ring. (A–C) Lateral views of excised, Alcian Blue/Alizarin Red-stained temporal bones from wild-type mice at post-natal days 5, 8, and 15. Grey dots highlight the tympanic ring dorsal edge at its apposition with the styloid process (sty). The endochondral styloid process is cartilaginous and stains blue at P5 and P8; the intramembranous tympanic ring (tr), is ossified at birth and stains red at all stages shown. s, stapes; i, incus; m, malleus. (D–F) Magnified views of the boxed regions in (A–C), respectively. Arrows indicate apparent vectors of tympanic ring growth; the tympanic ring is superficial to the styloid process and its expansion fully obscures the styloid process by P15. (G–I) show ventral views of the specimens shown in (A–C), respectively, with tympanic ring (tr) and malleus (m) dissected away for unobscured views of the stapes (s), facial canal (dotted line), and styloid process (sty). Note the near-complete ossification of the styloid process between stages P8 and P15. (TIF) [file pone.0109043.s002.tif]

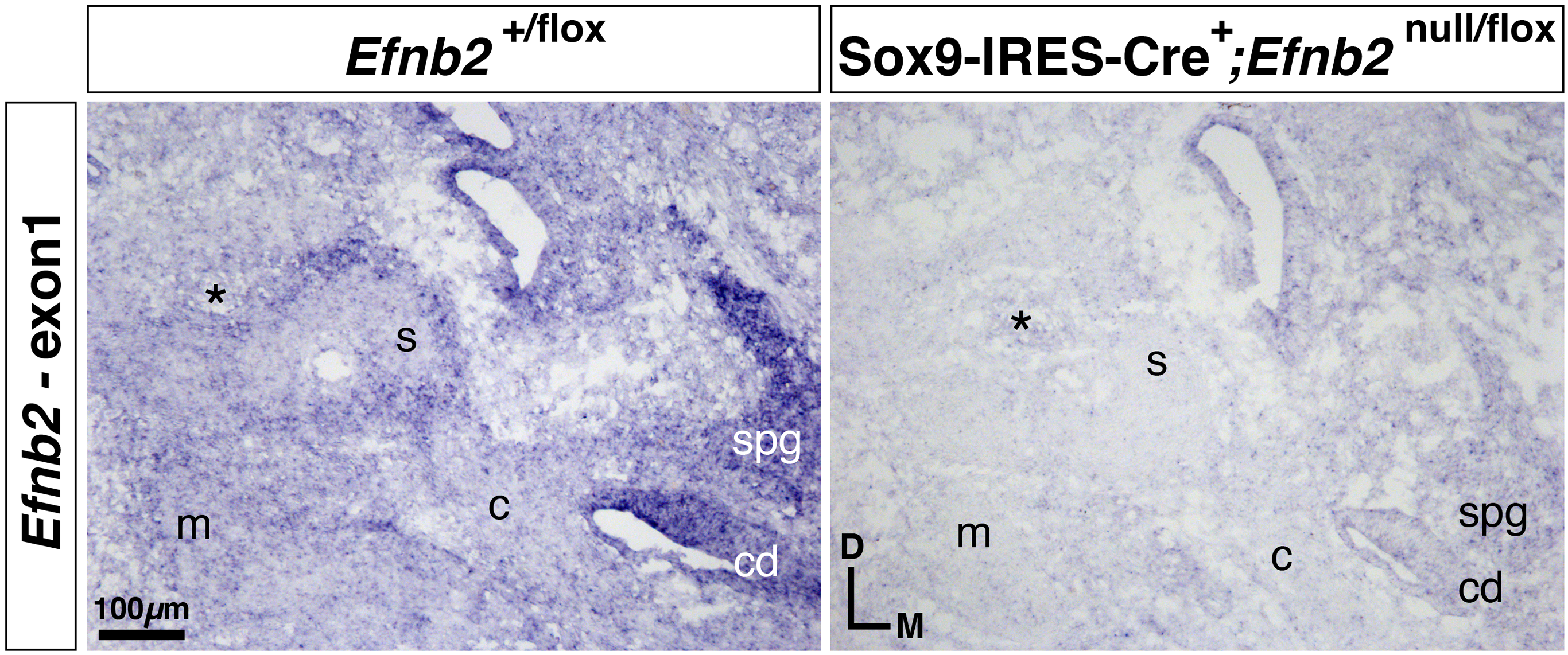

Supplement: Figure S3 — Validation of Sox9-IRES-Cre-mediated recombination at the Efnb2 locus. Sections of control (A) and Efnb2 CKO littermates (B), showing the middle ear at stage E14.5, hybridized with an Efnb2 exon1-specific probe. Signal from the Efnb2 exon1 probe in developing ear and second branchial arch tissues is markedly decreased in the mutant compared to control. asterisk, VIIth nerve; s, stapes; m, malleus; c, otic capsule; cd, cochlear duct; spg, spiral ganglion. (TIF) [file pone.0109043.s003.tif]

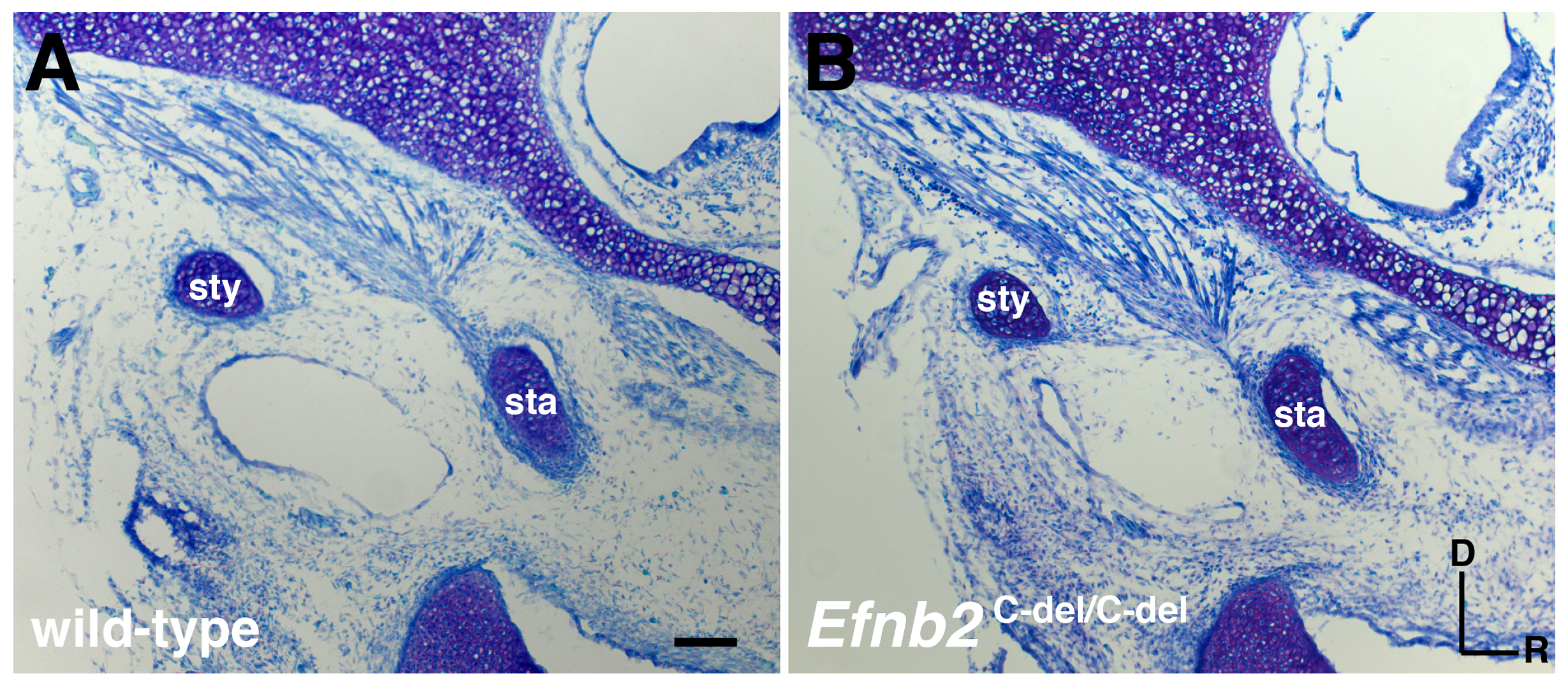

Supplement: Figure S4 — Homozygous deletion of the Efnb2 C-terminus has no apparent effect on stapes and styloid process morphology. Sagittal sections of mixed 129/CD1 strain wild-type control (A) and Efnb2 C-del/C-del (B) littermates at stage E18.5, stained with Toluidine Blue to reveal cartilage. Distance between the stapes (sta) and styloid process (sty) is similar across genotypes. Scale bar = 100 micrometers. (TIF) [file pone.0109043.s004.tif]

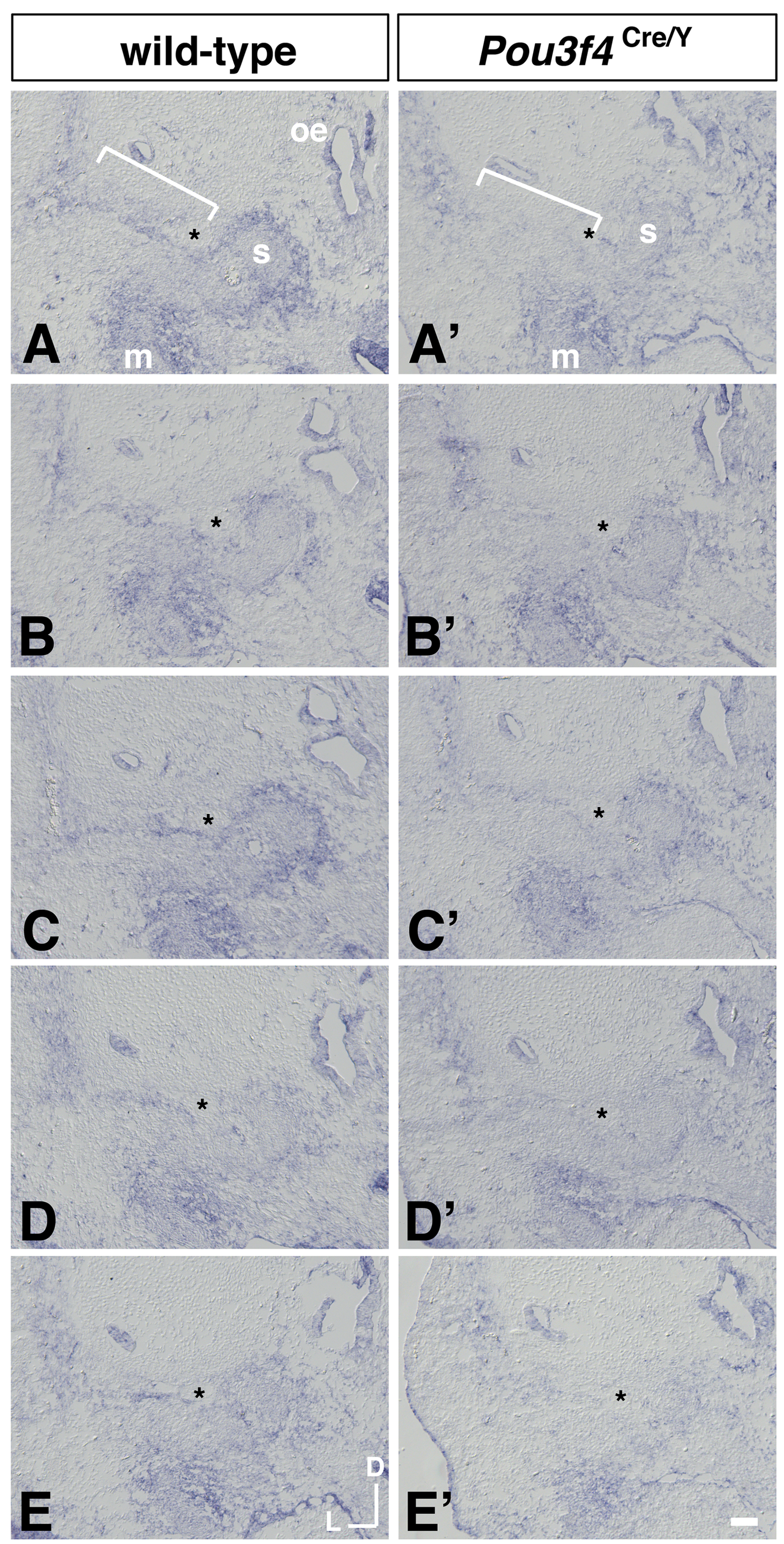

Supplement: Figure S5 — Efnb2 mRNA signal intensity is attenuated relative to control in Pou3f4 Cre/Y mutant mesenchyme dorsal to the stapes at stages E13–13.5. Representative image data for 5 of 6 wild-type (A–E) and mutant (A′–E′) littermate pairs, hybridized under controlled conditions to assay for potential change in Efnb2 expression across genotypes. Brackets in (A, A′) highlight mesenchyme dorsal to the stapes (s) and surrounding the VIIth nerve (asterisk), where Efnb2 hybridization signal is specifically altered across genotypes. Structures are identically framed in all panels. Efnb2 signals at the malleus (m) and otic epithelium (oe) appear similar across genotypes. Scale bar = 100 micrometers. The fifth of six wild-type/mutant pairs analyzed is shown in the main body of the text (Fig. 8A, A′, B, B′). (TIF) [file pone.0109043.s005.tif]

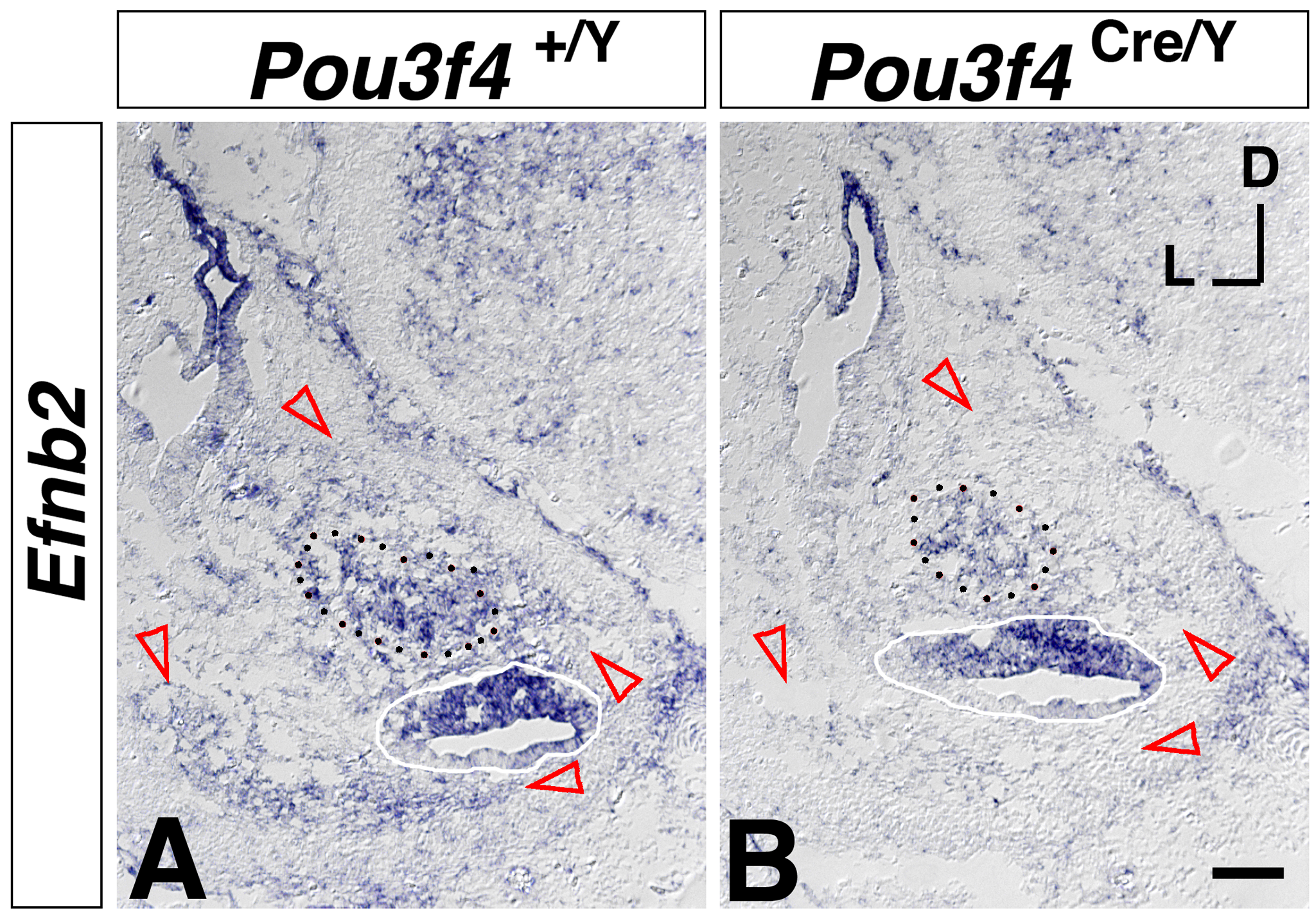

Supplement: Figure S6 — Efnb2 mRNA signal intensity is attenuated relative to control in Pou3f4 Cre/Y mutant sub-capsular mesenchyme at stage E13. Transverse sections of control (A) and Pou3f4 Cre/Y (B) E13.0 littermates hybridized to detect Efnb2. Red arrowheads highlight attenuation of Efnb2 hybridization signal in mutant sub-capsular mesenchyme surrounding the cochlea and spiral ganglion. Spiral ganglia are bounded by black dotted lines; cochleae are encircled by solid white lines. Scale bar = 100 micrometers. (TIF) [file pone.0109043.s006.tif]

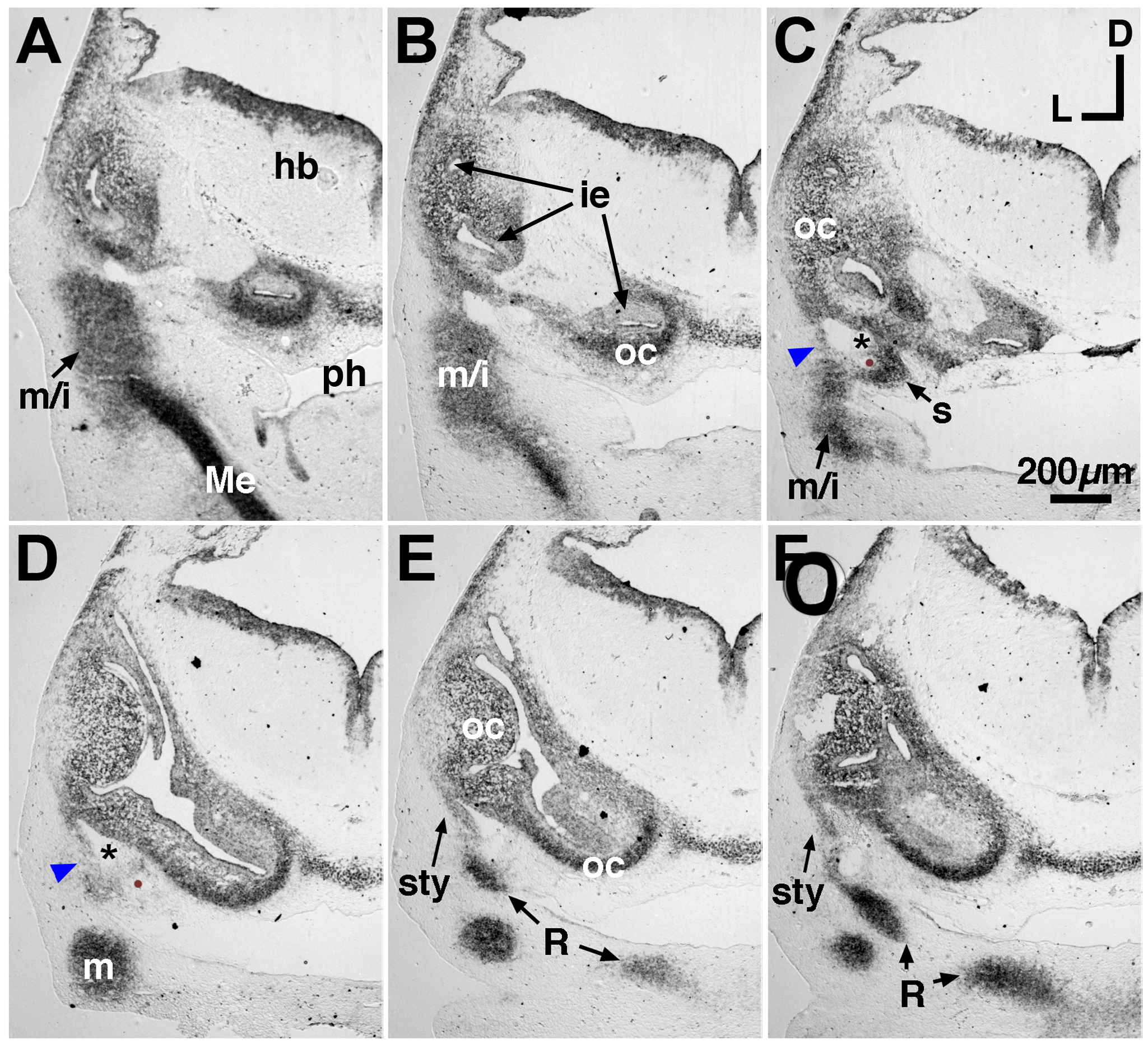

Supplement: Figure S7 — Sox9 marks a continuous domain comprising branchial arch cartilages, ossicles, styloid process, and otic capsule at stage E12.5. (A–F) Selected images from serial transverse sections through the 1st and 2nd branchial arches of an E12.5 embryo that were hybridized to detect Sox9 mRNA. Images are arranged in an anterior to posterior sequence (A through F). Note that Sox9 expression bridges all otic and branchial arch rudiments specified. Meckel's rudiment (Me), a dark-staining bar in (A) is cartilaginous at this stage; all other rudiments are mesenchymal condensations. Blue arrowheads in (C,D) highlight Sox9 signal bridging the stapes (s) and styloid process (sty). Asterisks in (C,D) highlight the VIIth cranial nerve. m/i, malleus/incus condensation; oc, otic capsule condensation; m, caudal end of the malleus/incus condensation in the 2nd arch; R, Reichert's cartilage rudiment, ie, inner ear; hb, hindbrain; ph, pharynx. (TIF) [file pone.0109043.s007.tif]
