# Supplementary material for: Pou3f4-Mediated Regulation of Ephrin-B2 Controls Temporal Bone Development in the Mouse
Source: PLoS One. 2014 Oct 9;9(10):e109043. doi: 10.1371/journal.pone.0109043 (PMC4192298; doi:10.1371/journal.pone.0109043)
Supplement: Table S1 — Absolute frequency and prevalence values for all possible phenotypic categories of stapes-styloid/facial canal connection in Pou3f4 Cre/Y and Efnb2 mutant samples. Sample number (n) refers to the number of mice analyzed. (PDF) [file pone.0109043.s008.pdf]

Table S1. Absolute frequency and prevalence values for all possible phenotypic categories (combinations of soft and/or bony/cartilaginous stapes defects) in *Pou3f4*$^{Cre/Y}$ and *Efnb2* mutant samples.

Genotype/Stage	*n*	Normal	Uni;Soft	Uni;B/C	Bilat;Soft	Soft and B/C	Bilat;B/C
Pou3f4$^{Cre/Y}$ adult	20	2 (.10)	4 (.20)	1 (.05)	6 (.30)	2 (.10)	5 (.25)
Pou3f4$^{Cre/Y}$ neonate	11	2 (.18)	2 (.18)	1 (.09)	2 (.18)	1 (.09)	3 (.27)
Efnb2 CKO fetal (E19)	10	1 (.10)	0	2 (.20)	0	2 (.20)	5 (.50)
Pou3f4$^{Cre/Y}$; *Efnb2*$^{null/flox}$ * #	20	3 (.15)	1 (.05)	9 (.45)	0	0	7 (.35)

Uni, unilateral; Soft, soft tissue bridge; B/C, bone or cartilage bridge; Bilat, bilateral. Category 'Soft and BC' denotes unilateral soft tissue bridge and a unilateral bone or cartilage bridge.

* P = 0.0419, compared to an expectation of equal frequencies for all categories, Chi-square: 11.53, 5 df. Statistical significance set at P < 0.05.

# P > .7232, where unilateral and bilateral bone-bridge frequencies are compared to an expectation of equal frequencies for unilateral and bilateral bone-bridges, Chi-square: 0.1255, 1df; or Fisher's exact test.
